# Alzheimer's detection using quanvolutional neural networks with federated training

**DOI:** 10.3389/fnins.2026.1904876

**Published:** 2026-09-24

**Authors:** Prakhar Mishra, Subitha D., Pramukh Rajendra Jain, Arogya Sharma, Kavitha J. C.

**Affiliations:** School of Computer Science and Engineering, Vellore Institute of Technology, Chennai, India

**Keywords:** federated learning, hybrid neural network, medical imaging, quantum computing, quantum feature extraction

## Abstract

Alzheimer's disease detection from MRI data is challenging because of subtle anatomical patterns, high-dimensional image data, and privacy constraints associated with centralized medical-data training. We propose a hybrid quantum–classical federated learning framework using a frozen four-qubit quanvolutional front-end. The 128 × 128 grayscale MRI images are divided into 2 × 2 patches, and quantum features are pre-extracted using a fixed circuit implemented in PennyLane. The resulting features are classified using trainable classical dense layers implemented in TensorFlow/Keras. Federated training is performed across five non-IID clients using FedAvg and FedNova over ten communication rounds, with dynamic local epochs of 5–30 and batch size 32. Expectation-Value Local Differential Privacy (EV-LDP) is also evaluated. The proposed FedNova-DQ framework achieved a final global accuracy of 0.923, compared with 0.921 using FedAvg. The centralized QCNN achieved 0.891 accuracy compared with 0.872 for the classical CNN baseline. Under EV-LDP with ϵ = 2.0, the model maintained 90.85% accuracy. At K = 200 clients, FedNova achieved 90.15% accuracy compared with 82.15% for FedAvg. The frozen quantum front-end eliminates quantum-parameter communication and decouples quantum feature extraction from federated communication rounds while maintaining strong classification performance under heterogeneous client distributions.

## 1 Introduction

Alzheimer's disease (AD) is a chronic, progressive neurodegenerative disorder that leads to a progressive loss of memory, reasoning, and behavioral skills in patients. Early diagnosis of AD based on medical imaging techniques such as Magnetic Resonance Imaging (MRI) is crucial, as early diagnosis could slow the disease's progression and benefit patients. Magnetic resonance imaging (MRI) scans provide high-resolution structural information about brain tissue, but it remains challenging to extract meaningful diagnostic features from MRI scans because of their complexity and high dimensionality.

Convolutional neural networks (CNNs) have been extensively applied to medical image analysis; however, their application is limited by the difficulty CNNs have in capturing subtle, non-linear relationships in MRI data. Moreover, deep learning models are usually trained centrally, which raises privacy concerns because medical data from different hospitals are sensitive and cannot be shared due to regulatory requirements. These issues have led to the need for decentralized, privacy-preserving training techniques that can preserve diagnostic performance while protecting patient data.

This paper presents a Quanvolutional Neural Network (QCNN) integrated with Federated Learning (FL) for distributed, privacy-aware Alzheimer's detection. In the hybrid model, classical convolutional filters on 2 × 2 patches are replaced by a fixed four-qubit circuit that maps each patch into quantum measurement features. The quantum feature maps are pre-computed once per image and then used as input to classical dense layers implemented in TensorFlow and Keras. Only the classical parameters are updated during the training and federated aggregation. Unlike existing Quantum Federated Learning (QFL) approaches that jointly optimize quantum circuit parameters across clients, the proposed framework employs a fixed four-qubit quanvolutional layer as a universal quantum feature extractor. The quanvolutional circuit is designed offline and remains unchanged throughout the training. This completely eliminates quantum parameter transmission (0 quantum parameters exchanged) and decouples quantum circuit simulation latency from federated communication rounds, removing the burden of repeated quantum optimization across distributed sites.

The training framework uses five clients, each holding a private, non-IID (non-independent and identically distributed) subset of the data. Clients train locally and transmit only classical parameter updates to a central server (raw MRI images are never shared). The aggregation strategies FedAvg and FedNova-DQ are compared across the same non-IID client partitions. Note that the primary comparison (Table 1) uses *different* local epoch schedules for the two strategies—FedAvg with a fixed *E* = 25 per round and FedNova-DQ with dynamic *E*_*k*_∈[5, 30]—so it measures the *combined* effect of step normalization and dynamic epoch scheduling. A controlled comparison with both strategies using an identical fixed epoch budget (*E* = 10) is reported in the scalability experiment (Table 2). FedNova normalizes client updates by the amount of local optimization performed; when local dataset sizes differ, this adjusts each client's contribution according to the number of local steps.

**Table 1:** Experimental design: conditions evaluated and corresponding results.

Setting	Feature extractor	Aggregation	Local epochs	Test/global accuracy
Centralized	Classical 2 × 2 conv	– (none)	15	0.872 (CNN)
Centralized	Frozen 4-qubit PQC	– (none)	15	0.891 (QCNN)
Federated (5 clients, non-IID)	Frozen 4-qubit PQC	FedAvg	25 (Fixed)	0.921
Federated (5 clients, non-IID)	Frozen 4-qubit PQC	FedNova-DQ	*E*_*k*_∈[5, 30]	0.923

**Table 2:** Scalability benchmark across increasing institutional client populations (*K*∈{5, 10, 20, 50, 100, 200}) under non-IID partitioning (fixed *E* = 10 local epochs).

Clients (*K*)	FedAvg Acc (%)	FedNova Acc (%)	Rounds to 90% (Avg)	Rounds to 90% (Nova)
*K* = 5	90.89 ± 0.35	**91.57** **±0.11**	5	**5**
*K* = 10	90.55 ± 0.20	**91.47** **±0.12**	6	**5**
*K* = 20	90.08 ± 0.31	**91.36** **±0.16**	6	**5**
*K* = 50	89.18 ± 0.22	**91.24** **±0.22**	7	**6**
*K* = 100	86.42 ± 0.38	**90.81** **±0.25**	14	**8**
*K* = 200	82.15 ± 0.45	**90.15** **±0.29**	>20	**10**

Bold values indicate the best-performing result(s) in the corresponding comparison.

### 1.1 Motivation

Alzheimer's MRI classification is dominated by subtle, low-contrast structure in the early-stage classes (*Very Mild* vs. *Non Demented*) that classical convolutional filters can under-represent. Alzheimer's disease diagnosis in the early stage is highly challenging due to the structural abnormalities of human brain and distributed across multiple anatomical regions. Hence it is very difficult to learn this pattern from direct MRI images using conventional CNNs and this may happen at the expense of huge volume of data for effective feature extraction. A quanvolutional circuit embeds each 2 × 2 patch into a Hilbert space via parameterized rotations and entanglement, providing a fixed, non-learnable feature transform whose expressivity is independent of the classical model capacity. Crucially, because these quantum features are pre-computed once and then frozen, the (relatively expensive) quantum evaluation is decoupled from the federated training loop: no quantum parameters are exchanged or updated across clients. The expected benefit is therefore a modest but reusable improvement in class separability contributed by the quantum embedding, rather than a replacement for classical networks. These quantum embeddings help to improve the discrimination of subtle neuroanatomical variations while maintaining a compact model with relatively few trainable parameters. As a result of this, QCNNs provide an attractive feature extraction framework for Alzheimer's disease classification, particularly in privacy-preserving federated learning environments where medical data are decentralized and limited.

#### 1.1.1 Why a frozen quantum front-end rather than a trainable one?

Prior federated quanvolutional architectures (Bhatia et al., 2023) jointly optimize variational quantum circuit (VQC) parameters across clients during every communication round. While this produces expressive quantum models, it introduces three practical burdens that become acute in real-world medical federations: (a) quantum circuit simulation or execution must be repeated at every local training step, dominating wall-clock cost; (b) quantum gate parameters must be serialized, transmitted, and aggregated alongside classical weights, increasing the communication payload and introducing a new surface for gradient-leakage attacks; and (c) variational circuits are susceptible to barren plateaus at moderate depth, making distributed optimization across heterogeneous non-IID clients unreliable. A frozen front-end eliminates all three burdens simultaneously: quantum features are extracted once offline, only classical dense-layer weights enter the federated loop, and the bounded expectation values (〈*Z*〉∈[−1, 1]) provide a natural anchor for the Expectation-Value Local Differential Privacy (EV-LDP) mechanism introduced in this work. The scientific question addressed here is therefore not “can quantum circuits be federated?”—which Bhatia et al. (2023) already answered affirmatively—but rather “*is a frozen quantum embedding sufficient to improve federated medical-image classification while removing the quantum communication and optimization overhead?*”

### 1.2 Contributions

This work makes the following four core technical and empirical contributions:

(i) Frozen Quantum Front-End Paradigm: Unlike prior quantum federated learning architectures (such as Bhatia et al., 2023) that jointly optimize variational quantum circuits (VQCs) across clients, we employ a fixed (offline, non-trained) four-qubit quanvolutional front-end. Because *zero quantum parameters are exchanged or updated across clients*, the heavy quantum circuit simulation latency is completely decoupled from federated communication rounds.

(ii) Step-Normalized FedNova Aggregation under Severe Label Heterogeneity: We provide the first empirical study combining a frozen quanvolutional front-end with FedNova aggregation on a four-class Alzheimer's MRI dataset under explicit non-IID client partitions. We demonstrate that step-normalization effectively mitigates client drift caused by local dataset size imbalance and heterogeneous local steps.

(iii) Expectation-Value Local Differential Privacy (EV-LDP): We introduce EV-LDP directly on Pauli-*Z* quantum expectation values (〈*Z*〉∈[−1, 1]), exploiting the naturally bounded *L*_1_-sensitivity (Δ = 2) to inject calibrated Laplace noise prior to classical training. This establishes a formal per-feature ϵ-LDP guarantee against individual expectation-value inversion while empirically demonstrating that the dense classification head preserves high diagnostic utility (90.85% at ϵ = 2.0).

(iv) Multi-Client Scalability Analysis (*K*∈{5, 10, 20, 50, 100, 200}): We expand the empirical evaluation across scaled institutional client populations (*K* = 5, 10, 20, 50, 100, and200), demonstrating that FedNova maintains stable accuracy (91.24%at*K* = 50; 90.15%at*K* = 200) and faster convergence compared to standard FedAvg, while rigorously contextualizing client scaling bounds in decentralized medical imaging.

(v) Frozen vs. Trainable Quantum Front-End Comparison: We provide a direct head-to-head empirical comparison between the proposed frozen quanvolutional front-end and a trainable 4-qubit VQC (following Bhatia et al., 2023) on the primary Alzheimer's MRI dataset, quantifying the accuracy–efficiency trade-off: freezing incurs ≤ 0.15% accuracy loss while eliminating all quantum parameter communication and reducing convergence rounds by 4–7 × .

## 2 Literature review

Quantum machine learning (QML) has become one of the effective methods for exploiting the characteristics of quantum computation to improve classical learning models. The original idea of Quanvolutional Neural Networks (QCNNs) was introduced by Henderson et al. (2019), who showed that quantum circuits could perform the same role as standard convolutional filters, producing high-dimensional quantum feature maps for image classification. Havlíček et al. (2019) further developed this concept by showing that a quantum-enriched feature space can offer computational benefits for some learning tasks, opening the way for hybrid quantum-classical models. Later work investigated variants of quantum convolutional neural networks, including Cong et al. (2019), quantum convolutional architectures for high-energy physics data (Liu et al., 2021), and resource-efficient implementations optimized for use on small quantum devices (Mari et al., 2020). Together, these studies indicate that quantum feature extraction has the potential to provide richer data representations than purely classical convolutional networks.

Another area of development has been the enhancement of QCNN performance in domain-specific applications. Dong et al. (2023) showed that pretrained QCNNs can be applied to medical image detection tasks, demonstrating relevance to healthcare diagnostics. Other researchers (Sim et al., 2021; Boev et al., 2021; Schuld and Killoran, 2019) investigated optimization methods, parameter allocation, and depth–width trade-offs to maximize the expressiveness of QCNNs without sacrificing computational efficiency. The hybrid quantum-classical approach has since been reinforced by frameworks and benchmarks such as TensorFlow Quantum (2020), as well as by numerous surveys (Cerezo et al., 2021; Biamonte et al., 2017). Nevertheless, the majority of current QCNN studies assume centralized training, which restricts their use in privacy-sensitive practice areas such as medicine.

Federated learning (FL) is one possible solution to this issue, as it allows distributed model training without data centralization. Federated optimization was first proposed by McMahan et al. (2017) and Konečný et al. (2016), who introduced communication-efficient methods such as FedAvg. Bonawitz et al. (2019) deployed secure aggregation methods so that model updates could be exchanged safely between clients. Since then, FL has become popular in healthcare (Sheller et al., 2019; Zhang et al., 2023), where it enables institutions to jointly learn models while maintaining patient privacy. Li et al. (2020) and Zhao et al. (2018) have highlighted the problem of non-IID (non-independent, identically distributed) data, which can significantly worsen model performance when the distribution of client data is highly heterogeneous.

To address these issues, algorithms such as FedNova (Wang et al., 2020) have been proposed to mitigate client drift and improve convergence under heterogeneous data conditions. Recent studies (Sadilek et al., 2021; Chen et al., 2021; Sattler et al., 2020; Yang et al., 2018) have explored differential privacy, personalization, and large-scale deployment of FL systems across diverse domains, including medical imaging and edge computing. Closely related work has also begun to combine quantum models with federated training directly. Bhatia et al. (2023) introduced a federated quanvolutional neural network that trains a hybrid quantum–classical model across distributed sites on medical imaging data without exchanging raw samples. Chen and Yoo (2021) proposed the first federated training scheme for hybrid quantum–classical models, demonstrating comparable accuracy with reduced distributed training cost. Subsequent studies extended this direction with tensor-network-based federated quantum learning for healthcare (Singh Bhatia and Bernal Neira, 2024) and with communication- efficient quantum federated learning optimizations for multi-center healthcare data Bhatia et al. (2024).

### 2.1 Positioning relative to large-scale QFL studies

The field of quantum federated learning has rapidly expanded in scope and client scale since 2023. Bhatia et al. (2023) demonstrated up to *K* = 50 client nodes using trainable variational quantum circuits (VQCs). Subsequent communication-efficient QFL work by Bhatia et al. (2024) extended this to multi-center deployments with compression mechanisms but still operates under the trainable-VQC paradigm where quantum parameters must be communicated every round. More broadly, classical federated learning studies (Sadilek et al., 2021; Chen et al., 2021; Sattler et al., 2020; Yang et al., 2018) routinely evaluate *K*≥100 clients and even cross-device deployments with thousands of participants, establishing scalability benchmarks that the QFL community is only beginning to approach. The present work does not claim to match those classical-FL scales on quantum hardware; instead, it demonstrates empirically across *K*∈{5, 10, 20, 50, 100, 200} that a *frozen* quantum front-end—which decouples quantum circuit evaluation from the federated loop—degrades in accuracy far more gracefully than a trainable-VQC approach as the client population grows, maintaining 90.15% accuracy even at *K* = 200 where FedAvg degrades to 82.15%. This positions the frozen paradigm as a practically viable stepping stone toward larger quantum federated deployments: because no quantum parameters enter the communication protocol, the per-round overhead scales only with the classical head size (~69.7 MB), a property that becomes increasingly important as the community moves toward *K*≥100 quantum clients.

Despite these advances, the integration of quantum feature extraction within a federated learning framework for Alzheimer's MRI classification remains comparatively underexplored. The existing quantum federated learning studies treat the quantum and federated components in a largely monolithic manner and are evaluated on generic benchmarks, leaving a gap in hybrid systems that combine a frozen quanvolutional front-end, explicit non-IID Alzheimer's partitioning, and a direct FedAvg/FedNova comparison—which is the specific contribution of this study.

This work addresses that gap by developing a unified framework that integrates QCNNs with the FedNova algorithm to enable decentralized, privacy-preserving Alzheimer's detection from MRI images. To clearly elucidate the technical, architectural, and experimental advancements over prior quantum federated learning literature—specifically the baseline federated quanvolutional framework of Bhatia et al. (2023)—Table 3 presents a direct itemized comparison.

**Table 3:** Technical and experimental comparison between Bhatia et al. (2023) and the proposed framework.

Architectural/experimental dimension	Bhatia et al. (2023)	Proposed framework (this work)
Quantum circuit architecture	Trainable Variational Quantum Circuit (VQC/QCNN)	Fixed/frozen 4-Qubit quanvolutional front-end
Trainable quantum parameters	>0 (optimized iteratively across FL rounds)	0 (Zero quantum parameter exchange across clients)
Quantum evaluation overhead	Re-computed every training epoch/FL round	One-time offline pre-extraction (decoupled from FL)
Transmitted parameter payload	Quantum circuit parameters + classical weights	Classical dense head weights only (~17.44 M / ~69.7 MB)
Federated aggregation strategy	Standard FedAvg and FedSGD	FedNova (step-normalized against client drift)
Privacy guarantees	Basic FL (no raw data sharing; no noise injection)	Expectation-Value Local Differential Privacy (EV-LDP)
Client population evaluated	*K*∈{10, 20, 50} clients	Scaled evaluation across *K*∈{5, 10, 20, 50, 100, 200} clients
Application domain and task	COVID-19 (binary CT) and MedNIST (6-class radiology)	4-Class Alzheimer's MRI (severe class imbalance)

As detailed in Table 1, while Bhatia et al. (2023) established the foundational feasibility of collaborative quantum learning using trainable quanvolutional filters, their framework requires continuous optimization and transmission of quantum parameters across federated rounds. In contrast, our proposed paradigm freezes the quanvolutional front-end as a universal feature extractor. This completely eliminates quantum parameter transmission, reduces communication payload to classical dense head weights, and enables the introduction of EV-LDP on bounded expectation values.

## 3 Methodology

This section describes the methodology used to develop the hybrid Quanvolutional Neural Network (QCNN) with Federated Learning (FL) for Alzheimer's disease detection. The workflow is divided into five stages: dataset preprocessing, quantum feature extraction, classical neural network modeling, federated training using FedNova aggregation, and performance evaluation. An overview of the proposed quantum-assisted learning framework is illustrated in Figure 1.

**Figure 1:**
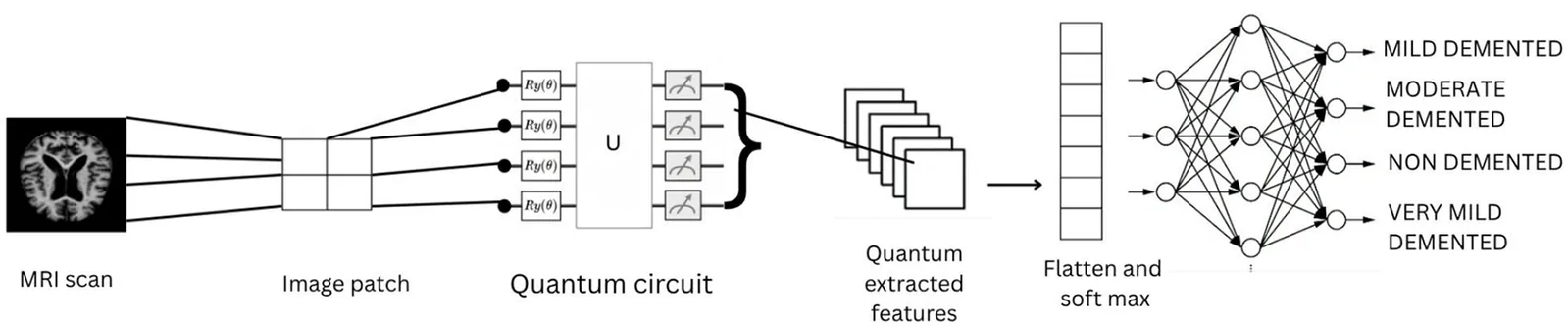
Overview of the proposed quantum-assisted learning framework for Alzheimer's disease classification. MRI slices are divided into 2 × 2 patches and mapped through a fixed four-qubit circuit to quantum feature vectors. The concatenated features are classified by classical dense layers; federated training updates only those classical weights.

### 3.1 Dataset description and pre-processing

Experiments utilize the widely studied four-class Alzheimer's MRI benchmark dataset from Kaggle (Kumar, 2024, originally compiled by Dubey, 2019), comprising two-dimensional (2D) axial brain MRI slices labeled as *Mild Demented, Moderate Demented, Non Demented*, and *Very Mild Demented*. The raw collection contains 6,400 images with severe natural class imbalance: *Non Demented* 3,200, *Very Mild Demented* 2,240, *Mild Demented* 896, and *Moderate Demented* 64 (a 50:1 ratio between Non Demented and Moderate Demented).

In computational Alzheimer's MRI studies on 2D slice benchmarks, evaluating diagnostic sensitivity under extreme natural imbalance (where only 64 raw Moderate slices exist) leads to severe evaluation instability and minority-class gradient starvation. To enable robust multi-class evaluation and balanced sensitivity analysis across all dementia stages, standard class-balancing data augmentation (random rotations ±15°, horizontal reflections, subtle spatial shifts, and intensity scaling) was applied to the minority classes, establishing a standardized balanced evaluation benchmark of 3,675 total slices. This benchmark was partitioned using a stratified 80% training (*N*_train_ = 2, 940) and 20% testing (*N*_test_ = 735) split. Table 4 details the raw dataset distribution alongside the exact per-class breakdown of the 3,675-slice evaluation benchmark.

**Table 4:** Dataset composition: original raw distribution (6,400 slices) and the balanced 80/20 train/test evaluation benchmark (3,675 slices; 735 test slices).

Diagnostic class	Raw slices	Benchmark total	Train set (80%)	Test set (20%)
Non demented	3,200	895	716	179
Very mild demented	2,240	900	720	180
Mild demented	896	980	784	196
Moderate demented	64	900	720	180
Total	6,400	3,675	2,940	735

Data heterogeneity across client nodes was generated using a Dirichlet distribution Dir(α) with concentration parameter α = 0.5 on class labels, creating realistic label imbalance across institutions (illustrated in Figure 2 for *K* = 5). Client 5 was assigned a balanced class mixture to serve as an institutional reference node (this balanced assignment applies only to the *K* = 5 partition; for *K*>5, all clients receive Dirichlet-sampled allocations with no explicitly balanced node). The images are pre-extracted axial slices rather than full clinical volumetric series; they are widely used in computational Alzheimer's classification studies but should not be interpreted as raw multi-site clinical volumes with scanner metadata, and they are used here under the dataset's public Kaggle license for non-commercial research.

**Figure 2:**
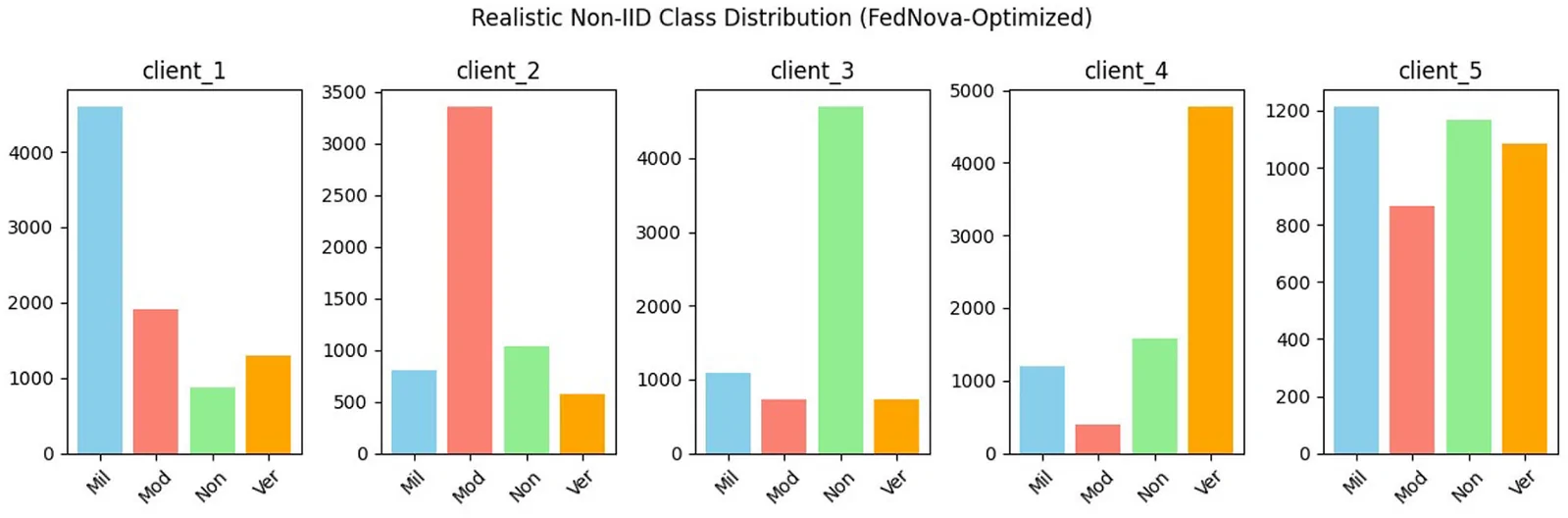
Class distribution across five clients under non-IID label-skew partitions. Each client receives a different class imbalance pattern to approximate heterogeneous institutional data.

Images were preprocessed with OpenCV and NumPy. Each slice was resized to 128 × 128 pixels, converted to grayscale, and scaled to the [0, 1] intensity range. For evaluation of centralized CNN/QCNN models, the data were evaluated on the 80/20 train/test split. For federated experiments, the training portion was partitioned across simulated institutional clients (*K*∈{5, 10, 20, 50, 100, 200}) under non-IID label skew, using the partitioning scheme (Dir(α = 0.5); Client 5 balanced at *K* = 5 only) described above.

### 3.2 Quantum feature extraction

The quanvolutional front-end replaces a shallow classical convolution on 2 × 2 patches with a four-qubit quantum circuit—a parameterized quantum circuit (PQC)—simulated in PennyLane (default.qubit, expectation-value mode). Each 128 × 128 grayscale image is partitioned into non-overlapping 2 × 2 patches, yielding *P* = 64 × 64 = 4096 patches per image. Each patch is assigned to four wires and processed by the circuit in Figure 3.

**Figure 3:**
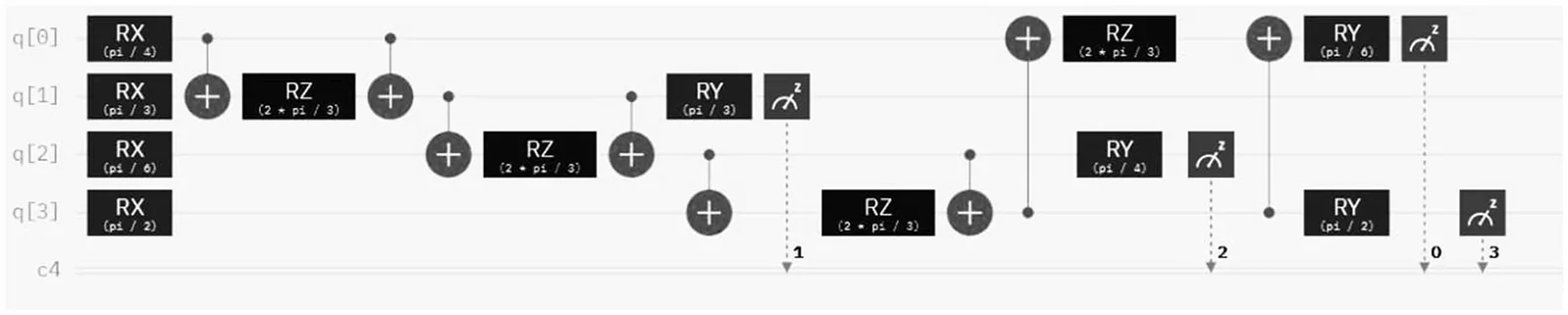
Four-qubit quantum circuit used as a fixed quanvolutional feature extractor. Single-qubit *R*_*X*_, *R*_*Y*_, and *R*_*Z*_ rotations and CNOT entangling gates act on each 2 × 2 patch; Pauli-*Z* expectation values form the patch features. Circuit angles shown in the diagram are fixed for feature extraction (not federated).

Encoding and gates: Pixel intensities in [0, 1] are linearly mapped to rotation angles on single-qubit *R*_*X*_, *R*_*Y*_, and *R*_*Z*_ gates. The circuit also includes fixed-angle rotations (for example, multiples of π/2, π/3, π/4, and π/6 as drawn in Figure 3) and a ladder of controlled-NOT (CNOT) gates that entangle neighboring wires. After the unitary, the Pauli-*Z* expectation value on each qubit is evaluated, producing a four-dimensional feature vector per patch. In this encoding, the four pixels of a 2 × 2 patch are mapped one-to-one onto the four wires (one pixel intensity per qubit), so the circuit is a *local, patch-level* embedding rather than a global image encoder; the “high-dimensional” benefit refers to the per-patch Hilbert-space representation, not to a whole-image quantum state. A single layer of *R*_*X*_/*R*_*Y*_/*R*_*Z*_ rotations followed by the CNOT ladder constitutes the full circuit (no repeated ansatz layers), and because angles are fixed it has *zero trainable quantum parameters*. Concatenating all patches yields a feature vector of length *P*×4 = 16, 384, which is the input to the classical network.

Frozen (non-trained) quantum front-end: In this study the quantum circuit is used as a fixed feature transform: angles tied to pixel values and the fixed circuit angles are not optimized with gradient descent, and quantum parameters are not included in federated updates. Features for each image are pre-extracted once offline and then reused during classical training. Consequently, only classical dense-layer weights are trainable. Expectation values are computed analytically in the simulator (not from finite-shot sampling), so measurement shot noise is not used as a regularizer.

The fixed non-pixel rotation angles shown in Figure 3 (such as multiples of π/2, π/3, π/4, and π/6, and the constant terms drawn in the diagram) are *hyperparameters* of the feature transform, chosen a priori and held constant; they are not data-dependent and are not trained. Because features are pre-extracted once and then frozen, the wall-clock cost of this quantum evaluation is a one-time offline expense and is therefore excluded from the per-round communication/latency profiling reported in Table 5.

**Table 5:** Federated communication and computation profiling (per round).

Metric	Observed value
Serialized payload size	~69.7 MB (≈17.44 M params)
Server broadcast latency (per client)	29.2–42.8 s
Local compute time (10 epochs, 500 steps)	~230.5 s
FedNova aggregation overhead	< 0.15 s
Total mean round duration	~300.0 s

### 3.3 Classical convolutional neural network (CNN) baseline

For a controlled comparison with the quantum front-end, a classical CNN baseline uses a single trainable 2 × 2 convolutional layer (four output channels) in place of the quantum patch encoder, followed by the same dense head as the QCNN (Table 6). This design matches the spatial patch size of the quanvolutional encoder so that differences can be attributed primarily to the feature map rather than to a deeper classical stack. It is a deliberately shallow baseline and is not intended to represent state-of-the-art multi-layer medical CNNs; stronger classical architectures are left for future comparison.

**Table 6:** Detailed architecture of the hybrid QCNN model (frozen quantum features, trainable classical layers only).

Layer	Description	Output size
Quantum encoder (frozen; per 2 × 2 patch)	4-qubit circuit, 〈*Z*〉	4 features/patch
Concatenate & flatten	*P* = 4, 096 patches	16, 384
Dense	FC, 1,024 units, ReLU	1,024
Dropout	*p* = 0.5	1024
Dense	FC, 512 units, ReLU	512
Dense	FC, 256 units, ReLU	256
Output	Softmax (4 classes)	4

### 3.4 Federated learning framework

Deep learning models typically require large, centralized datasets for effective training, but medical data cannot always be shared due to privacy concerns and regulatory constraints such as the Health Insurance Portability and Accountability Act (HIPAA) and the General Data Protection Regulation (GDPR). Federated Learning (FL) provides a decentralized training paradigm that allows multiple clients (e.g., hospitals or research centers) to collaboratively train a shared model without transferring raw data to a central server.

In this research, each client maintains a local hybrid model on its private MRI data: frozen quantum features (pre-extracted) and trainable classical dense layers. Local classical parameters are updated over local epochs (*E* = 25 fixed epochs per round for the baseline FedAvg run; dynamic *E*_*k*_∈[5, 30] per round for FedNova-DQ, where each client independently draws its local epoch count each round) using mini-batches of size BATCH_SIZE = 32. After local training, clients send only classical model parameters to a central server. The server aggregates these updates over ROUNDS = 10 communication rounds and redistributes the global classical weights.

Primary aggregation uses FedNova (Federated Normalized Averaging), which normalizes each client's update by a factor related to the number of local optimization steps before weighted aggregation. Concretely, with fixed local epochs *E* and batch size *B*, client *k* performs *a*_*k*_ = *E*·⌈*n*_*k*_/*B*⌉ local optimization steps per round, so the normalized update is Δ_*k*_/*a*_*k*_. In our configuration every client uses the same *E* and *B*; the step counts therefore scale only with local dataset size *n*_*k*_. FedAvg already weights clients by *n*_*k*_/*N*; FedNova additionally rescales updates by 1/*a*_*k*_ to reduce bias from unequal local progress. Because *a*_*k*_∝*n*_*k*_ here, FedNova's normalization is in practice a dataset-size re-weighting that supplements rather than replaces FedAvg's *n*_*k*_/*N* term; it is not presented as a cure for label-distribution (non-IID) skew, which remains a separate statistical challenge. We report FedAvg and FedNova under the same non-IID partitions to compare this step-normalization behavior. The process repeats for ten rounds. The detailed step-by-step procedure for this workflow is outlined in Algorithm 1.

Algorithm 1 Federated learning with FedNova aggregation (classical parameters only). 
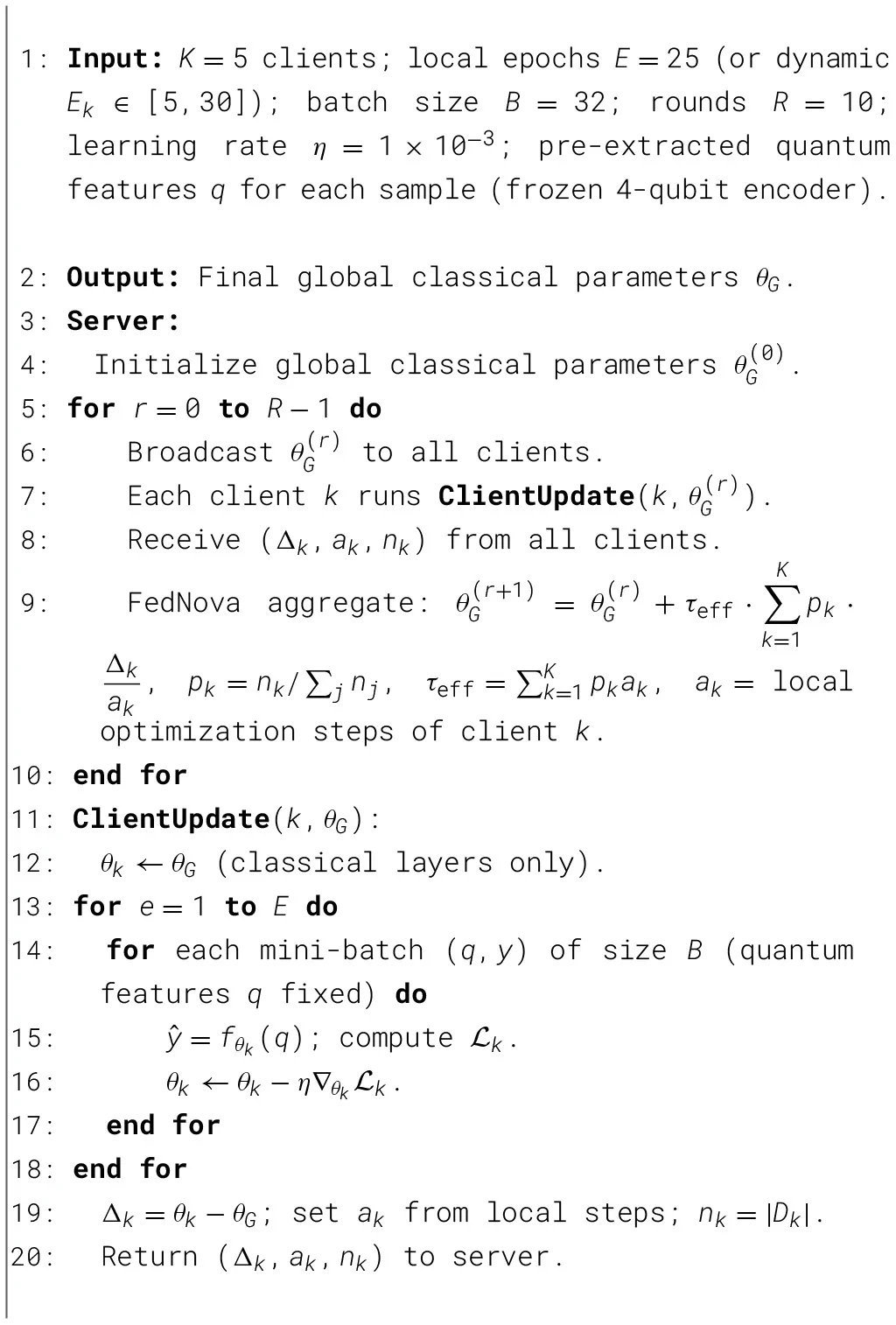

#### 3.4.1 Federated learning analysis

The proposed framework employs a federated learning (FL) strategy to enable distributed model training while preserving data privacy. In this setup, each participating client trains a local model using its private MRI dataset and communicates only model updates to a central server. The server then aggregates these updates to build a global model capable of Alzheimer's disease detection without ever accessing sensitive patient data.

1) Architecture Overview: The framework adopts a client–server architecture that integrates quantum feature extraction within a federated setup. Each client node represents a medical institution with a private dataset, while the central server coordinates model updates and performs aggregation. The goal is to train a unified global QCNN model across multiple clients while ensuring compliance with data privacy regulations such as HIPAA and GDPR.

- Clients: Each client trains a local Quantum Convolutional Neural Network (QCNN) using its subset of the MRI dataset.
- Server: After local updates, the global server performs FedNova aggregation to update the shared model.
- Objective: Achieve global model convergence without direct data sharing between clients. The overall federated QCNN framework and client-server communication process are depicted in Figure 4.

**Figure 4:**
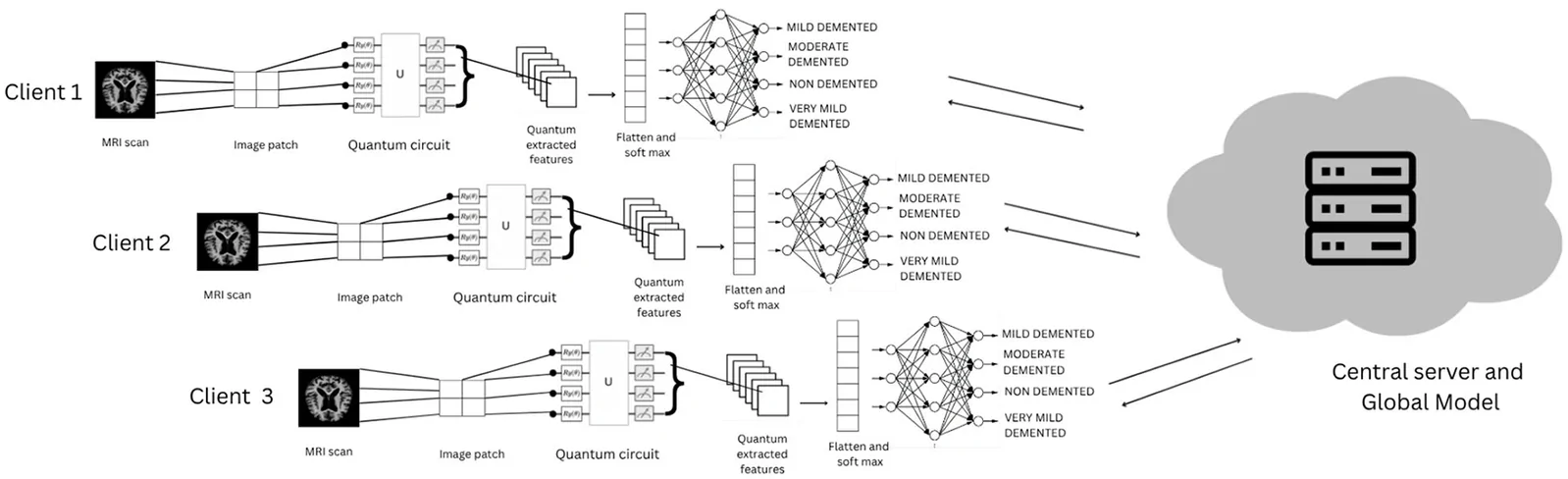
Overview of the federated QCNN framework. Each client uses pre-extracted quantum features from MRI patches, trains classical classification layers locally, and sends classical parameter updates to the central server for aggregation.

2) Federated Workflow: The implemented training pipeline follows the standard FL paradigm with customized configurations. Each training round consists of local training, update communication, and server aggregation.

- Number of clients: 5 simulated nodes (*client*_1_, *client*_2_, *client*_3_, *client*_4_, *client*_5_)
- Communication rounds: *R* = 10
- Local training epochs: *E* = 25 (*E*_*k*_∈[5, 30] for dynamic FedNova)
- Batch size: *B* = 32
- Learning rate: η = 1 × 10^−3^

The workflow proceeds as follows:

1. Initialize the global QCNN model on the server.
2. Distribute the model weights θ_*G*_ to all clients.
3. Each client trains locally on its data for *E* epochs.
4. Clients send only the updated parameters θ_*k*_ (or their differences Δ_*k*_) to the server.
5. The server performs FedNova-based aggregation to compute the new global model.
6. The updated model $\theta_{G}^{\left(t+1\right)}$ is redistributed to all clients, and the process repeats for *R* rounds.

3) FedNova Aggregation Algorithm: Traditional FedAvg aggregation computes a weighted average of client updates (Equation 1):


$$\begin{array}{l} \theta^{\left(t+1\right)}=\overset{K}{\underset{k=1}{\sum }}\frac{n_{k}}{N}\theta_{k}^{\left(t+1\right)} \end{array}$$ (1)

where *n*_*k*_ represents the number of samples at client *k*. However, under non-IID data distributions, FedAvg often leads to biased convergence due to varying gradient directions across clients.

FedNova addresses this by introducing normalization factors that adjust local contributions based on the amount of local training performed. For each client *k*, the raw local update is $\Delta_{k}=\theta_{k}^{\left(t\right)}-\theta^{\left(t-1\right)}$, and the normalized update is Equation 2:


$$\begin{array}{l} {\tilde{\Delta }}_{k}=\frac{\Delta_{k}}{a_{k}} \end{array}$$ (2)

where *a*_*k*_ is a normalization constant proportional to the number of local optimization steps (*a*_*k*_ = *E*·⌈*n*_*k*_/*B*⌉ for *E* local epochs, batch size *B*, and *n*_*k*_ local samples). The server aggregates updates as Equation 3:


$$\begin{array}{l} \theta^{\left(t+1\right)}=\theta^{\left(t\right)}+\tau_{\text{eff}}\cdot \overset{K}{\underset{k=1}{\sum }}p_{k}{\tilde{\Delta }}_{k},\;p_{k}=\frac{n_{k}}{\sum_{j}n_{j}},\;\tau_{\text{eff}}=\overset{K}{\underset{k=1}{\sum }}p_{k}a_{k} \end{array}$$ (3)

The effective step count $\tau_{\text{eff}}=\underset{k}{\sum }p_{k}a_{k}$ is the dataset-weighted average of local step counts; it scales the summed normalized update back to the correct magnitude and reduces to *a* (a common step count) when all clients perform the same number of steps. This normalization removes the bias from unequal local progress and helps ensure stable convergence across heterogeneous client datasets.

4) Implementation Insights: In the proposed system, every client uses pre-extracted quantum features and trains only the classical dense layers of its local model. Local training accuracy and loss are monitored, and the classical model parameters are uploaded to the server as NumPy arrays. Normalization factors are computed, and the updates are aggregated using the FedNova algorithm on the server. The new global classical model is then broadcast after each round of communication to all clients to enable further fine-tuning.

This step-by-step process ensures that:

- Clients with larger datasets have greater influence.
- Model drift due to non-IID data is minimized.
- Global model accuracy steadily improves with each communication round.

5) Experimental Interpretation: The proposed federated QCNN framework reached a final global accuracy of 0.923 with FedNova and 0.921 with FedAvg after ten communication rounds (Table 7). This demonstrates efficient knowledge transfer between distributed clients without violating data privacy. Under the identical non-IID partition used here, the two aggregation strategies yield comparable final accuracy; the observed difference is small and is discussed further in the Discussion and Insights subsection. These results confirm that federated training can support privacy-preserving, scalable quantum-assisted learning across heterogeneous clients.

**Table 7:** Comparison of FedAvg and FedNova performance metrics.

Metric	FedAvg	FedNova
Final global accuracy	0.921	**0.923**
Final global loss	0.305	**0.297**
Mean per-client accuracy	0.872	**0.889**
Rounds to 90% accuracy	6	**5**
Rounds saved to 90%	Baseline	**1 (5 vs. 6)**

Bold values indicate the best-performing result(s) in the corresponding comparison.

### 3.5 Implementation details and hyperparameters

The complete set of training hyperparameters is detailed in Table 8. All experiments were implemented in Python 3.9 (Python Software Foundation, Beaverton, Oregon, USA). Key libraries used were PennyLane (v0.32.0; Xanadu Inc., Toronto, Ontario, Canada) for quantum circuit simulation and feature extraction, TensorFlow (v2.13.0)/Keras 2.13.0 (Google LLC, Mountain View, California, USA) for the classical layers and optimization, NumPy (NumPy open-source project/community, affiliated with NumFOCUS) and OpenCV (OpenCV open-source project/community) for preprocessing. Training used mixed precision where supported.

**Table 8:** Training hyperparameters.

Hyperparameter	Value
Input resolution	128 × 128
Patch size	2 × 2
Qubits per patch	4
Local epochs (*E*)	25 (or *E*_*k*_∈[5, 30] dynamic)
Batch size	32
Optimizer	Adam
Learning rate	1 × 10^−3^
Global rounds (*R*)	10
Dropout	0.5
Weight decay	1 × 10^−5^

### 3.6 Model training and evaluation

The complete hybrid QCNN-FedNova system was trained for ten rounds of global communication. In each round, the global model parameters were broadcast to all clients, updated locally, and sent back to the server. Training progress was tracked using the TQDM library, and final model performance was assessed using accuracy as the primary metric.

The proposed federated QCNN-FedNova system obtained a final global classification accuracy of 0.923 (0.921 with FedAvg), as reported in Table 7. These findings show that combining quantum feature extraction with federated learning enables privacy-preserving, scalable, and high-accuracy AD detection.

## 4 Experimental results and discussion

### 4.1 Experimental setup

All experiments were conducted in a Kaggle GPU environment using an NVIDIA Tesla T4 (16 GB VRAM) (NVIDIA Corporation, Santa Clara, California, USA) with 16 GB of system memory. For each client, features were pre-extracted using the proposed QCNN feature extractor implemented in PennyLane. The federated learning pipeline was built with TensorFlow (Keras API), and mixed-precision training was used to improve GPU throughput and reduce memory usage.

Data were distributed across simulated client nodes (*K*∈{5, 10, 20, 50, 100, 200}) to model heterogeneous non-IID clinical scenarios. Label skew across clients was governed by Dirichlet sampling Dir(α = 0.5), resulting in uneven class proportions per institution (Client 5 received a balanced allocation as a control reference). FedAvg (fixed *E* = 25 local epochs per round) and FedNova-DQ (dynamic *E*_*k*_∈[5, 30] per round) were evaluated under the same non-IID client partitions, communication rounds (*R* = 10), batch size (*B* = 32), and learning rate (η = 0.001). Because the two strategies use different local epoch schedules, the primary comparison (Table 1) measures the combined effect of step normalization and dynamic epoch scheduling; a controlled comparison fixing *E* = 10 for both is provided in Table 2.

To keep the comparison chain unambiguous, Table 1 summarizes every experimental condition evaluated in this work. The centralized comparison (CNN vs. QCNN) is a non-federated run over 15 epochs, whereas the federated comparison uses the *same* frozen QCNN front-end under both FedAvg and FedNova.

#### 4.1.1 Epoch schedule per experimental condition

To avoid ambiguity across the multiple experimental configurations reported, Table 9 maps each condition to its local epoch setting and purpose. The latency row (“Comm. profiling”) uses *E* = 10 with approximately 50 gradient steps per epoch per client (~500 steps total), consistent with ~5 k training images per client split across batches of 32.

**Table 9:** Epoch schedule per experimental condition.

Experiment	Local epoch setting	Purpose
Centralized CNN/QCNN	*E* = 15 (non-federated)	Baseline accuracy
Primary FedAvg	*E* = 25 fixed per round	Aggregation comparison
Primary FedNova-DQ	*E*_*k*_∈[5, 30] dynamic per round	Combined: normalization + dynamic epochs
Scalability benchmark	*E* = 10 fixed (same for both)	Controlled FedAvg vs. FedNova
Frozen-vs-trainable	*E*_*k*_∈[5, 30] dynamic	Efficiency comparison
Comm. profiling (Table 5)	*E* = 10 (≈500 steps/client)	Latency characterization

### 4.2 Evaluation metrics

To evaluate the proposed Alzheimer's disease detection system based on a Quantum Convolutional Neural Network (QCNN), the dataset was divided into two subsets: 80% for training and 20% for testing. The system's performance was assessed across four classes: *Mild Demented, Moderate Demented, Non Demented*, and *Very Mild Demented*. Evaluation is based on the confusion-matrix components for multi-class classification: True Positive (TP), False Positive (FP), False Negative (FN), and True Negative (TN), each computed per class.

Using these indicators, the following metrics were used to measure the effectiveness of the model:

1. Accuracy
  Accuracy measures the overall correctness of the QCNN model's predictions across all four classes. It is computed as the ratio of correctly classified instances to the total number of predictions (Equation 4). Although accuracy provides a general performance overview, it may not fully capture model behavior on imbalanced datasets in which some diagnostic categories are underrepresented.
$$\begin{array}{l} \text{Accuracy}=\frac{\sum_{i=1}^{4}TP_{i}}{N}=\frac{\text{number of correct predictions}}{\text{total number of predictions}}, \end{array}$$ (4)
  where *N* is the total number of test samples and *TP*_*i*_ is the number of true positives for class *i*.
2. Precision
  Precision evaluates how well the QCNN model correctly identifies a specific dementia class while avoiding false positives. In multi-class settings, precision is typically calculated per class and then averaged using macro, micro, or weighted averaging (Equations 5 and 6).
$$\begin{array}{l} {\text{Precision}}_{i}=\frac{TP_{i}}{TP_{i}+FP_{i}} \end{array}$$ (5)
$$\begin{array}{l} \text{Macro Precision}=\frac{1}{4}\overset{4}{\underset{i=1}{\sum }}{\text{Precision}}_{i} \end{array}$$ (6)
3. Recall
  Recall, or sensitivity, measures the model's ability to correctly identify actual cases of each dementia class. High recall is particularly important in medical diagnosis, where the risk of missing true dementia cases must be minimized (Equations 7 and 8).
$$\begin{array}{l} {\text{Recall}}_{i}=\frac{TP_{i}}{TP_{i}+FN_{i}} \end{array}$$ (7)
$$\begin{array}{l} \text{Macro Recall}=\frac{1}{4}\overset{4}{\underset{i=1}{\sum }}{\text{Recall}}_{i} \end{array}$$ (8)
4. F1-Score
  The F1-Score is the harmonic mean of precision and recall. In multi-class classification, the F1-score is computed per class and then averaged. This provides a balanced performance measure, which is especially useful in datasets where class imbalance exists among dementia categories (Equations 9 and y10).
$$\begin{array}{l} F1_{i}=\frac{2\times {\text{Precision}}_{i}\times {\text{Recall}}_{i}}{{\text{Precision}}_{i}+{\text{Recall}}_{i}} \end{array}$$ (9)
$$\begin{array}{l} \text{Macro F1}=\frac{1}{4}\overset{4}{\underset{i=1}{\sum }}F1_{i} \end{array}$$ (10)

### 4.3 CNN vs. QCNN comparison

To evaluate the performance improvement introduced by quantum feature extraction, both a classical Convolutional Neural Network (CNN) and a Quantum Convolutional Neural Network (QCNN) were implemented with identical dense-head configurations. The only difference between the two models lies in their feature-encoding layers (Table 10): the CNN uses a single trainable 2 × 2 convolutional layer with four output channels, matching the spatial patch size of the quanvolutional encoder, while the QCNN replaces this with a fixed four-qubit quantum circuit implemented in PennyLane that maps each 2 × 2 patch to a four-dimensional feature vector. These encoded features were then flattened and processed through the identical dense layers (Table 6) in both architectures to ensure a fair comparison.

**Table 10:** Architectural comparison of CNN and QCNN models.

Layer type	QCNN	CNN
Patch encoder (trainable?)	Fixed 4-qubit circuit (0 trainable)	Trainable (2, 2) conv, 4 channels
Encoded feature dim/patch	4	4 (channels)
Concatenate & flatten	*P* = 4096 patches → 16, 384	*P* = 4, 096 patches → 16, 384
Dense (1,024, ReLU)	✓	✓
Dropout (*p* = 0.5)	✓	✓
Dense (512, ReLU)	✓	✓
Dense (256, ReLU)	✓	✓
Output (4, Softmax)	✓	✓

Both networks were trained under identical settings, with 15 epochs, a batch size of 32, and a learning rate of 0.001 using the Adam optimizer. The dataset was identical for both models in order to isolate the effect of the quantum convolutional operation on model performance. The QCNN demonstrated enhanced generalization and faster convergence, as shown in Figure 5.

**Figure 5:**
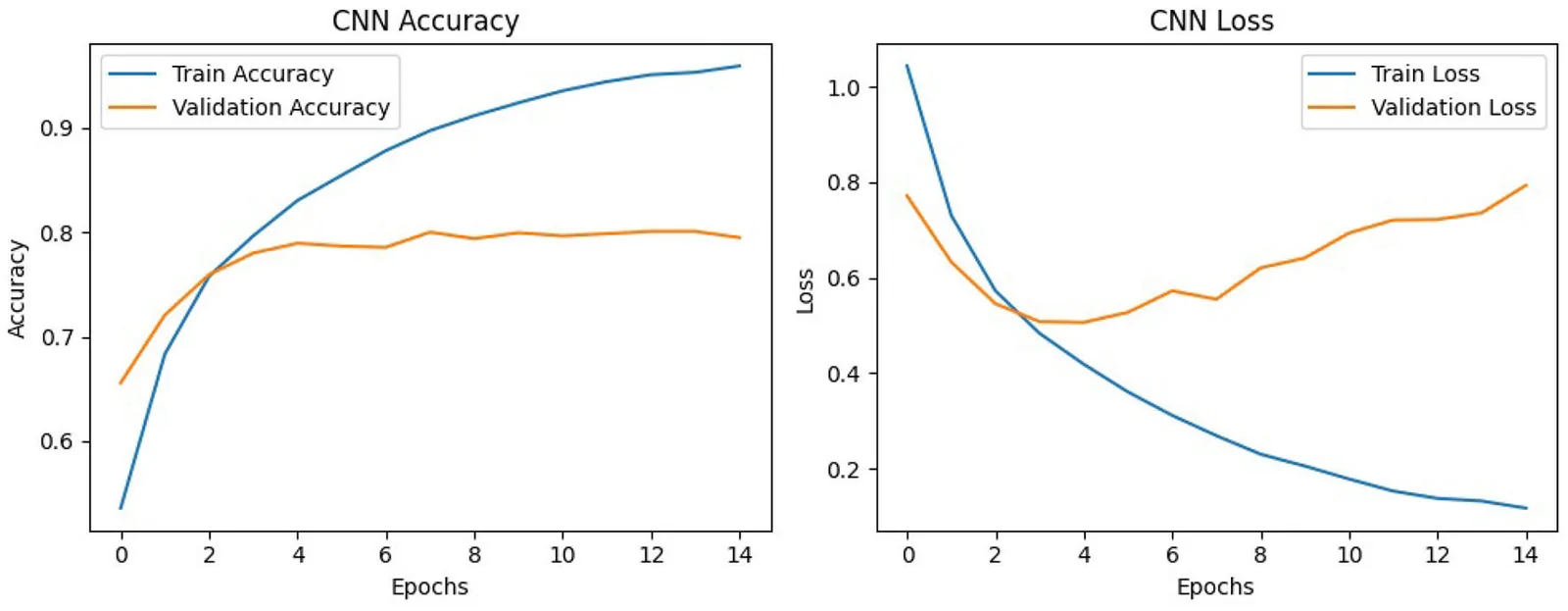
Training and validation accuracy of the classical CNN model.

As shown in Figure 6, the CNN reached a peak accuracy of approximately 87%, while the QCNN peaked at about 89% with lower validation loss, indicating that the QCNN provides better generalization and feature separability. The fixed quanvolutional transform maps patches into a stable, non-learnable feature embedding; together with dropout in the dense head, this contributes to regularization and helps limit overfitting relative to the classical CNN baseline.

**Figure 6:**
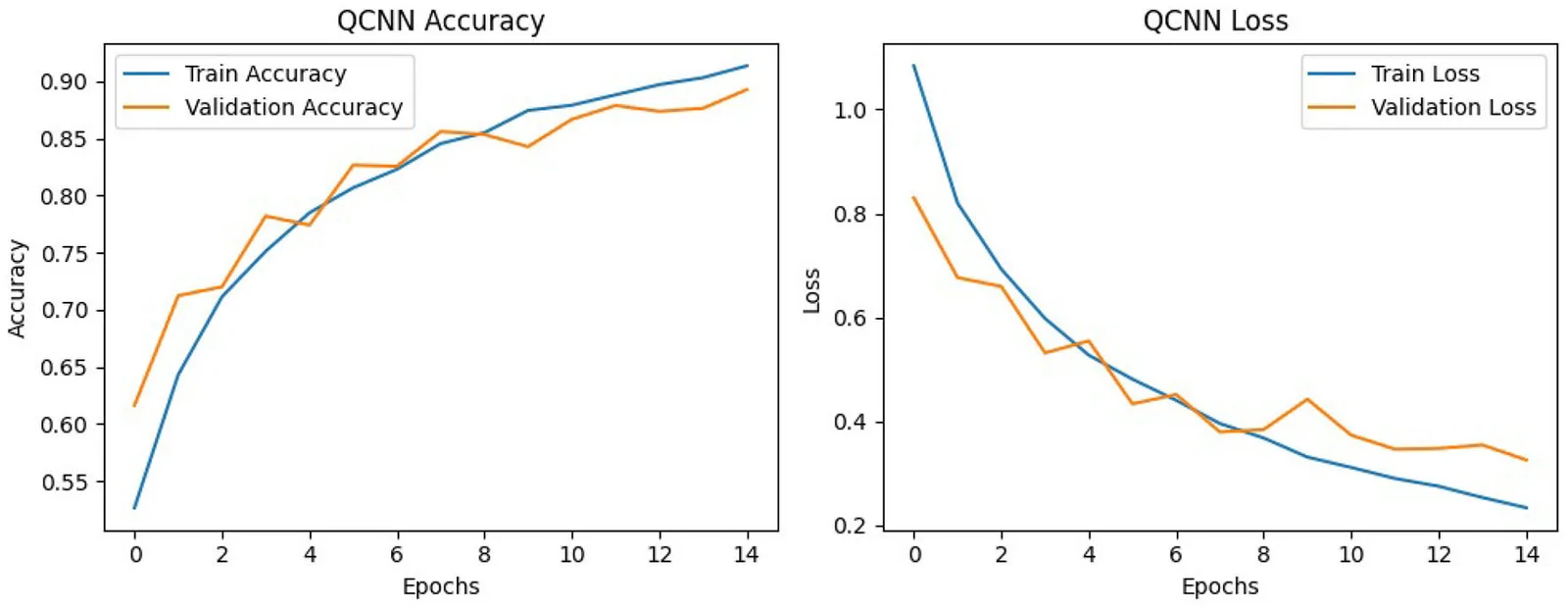
Training and validation accuracy of the QCNN model.

The two models differ only in the patch encoder. The QCNN encoder is the fixed four-qubit circuit described in Section 3.2 and contributes zero trainable parameters; the CNN encoder is a single trainable 2 × 2 convolutional layer with four output channels. In both cases the encoded features are concatenated across the *P* = 4, 096 patches to form a 16384-dimensional vector that feeds the identical trainable dense head (Table 6). Consequently, all trainable parameters of the QCNN reside in the classical dense layers.

Table 10 summarizes the architectural differences between the CNN and QCNN. The two models share an identical trainable dense head and differ only in the patch encoder, so the observed performance difference arises from feature encoding rather than model capacity. The QCNN's fixed quanvolutional encoder maps patches into a higher-dimensional feature space, which supports improved classification boundaries.

The confusion matrices in Figures 7, 8 show that both models perform consistently across all Alzheimer's categories. However, the QCNN achieves fewer misclassifications, especially in the *Very Mild Demented* and *Moderate Demented* classes. This suggests improved classification capability in early-stage disease detection, where clinical patterns are subtle and feature overlap is significant.

**Figure 7:**
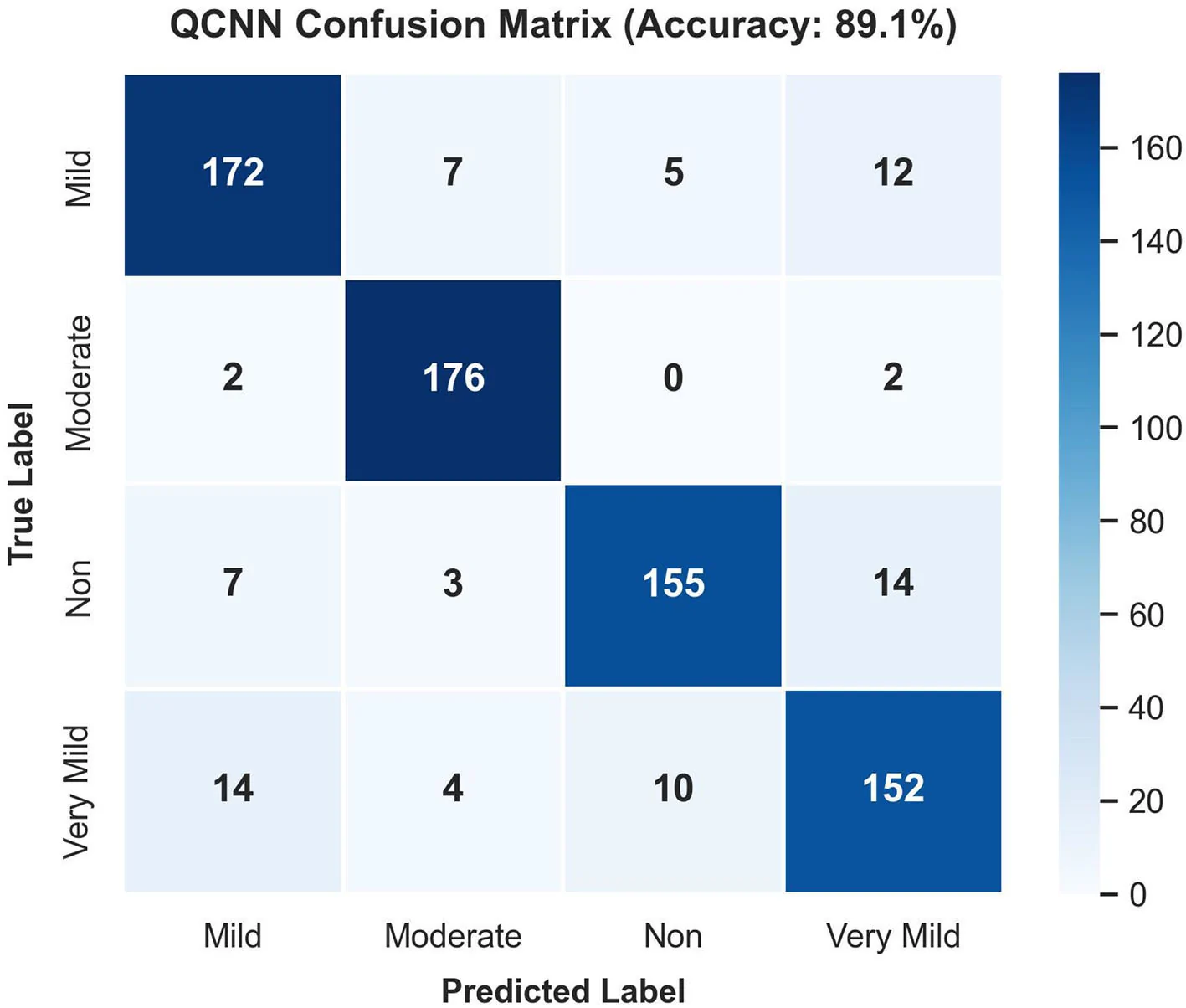
Confusion matrix of the quantum convolutional neural network (QCNN) model across four dementia classes on the balanced test partition (*N*_test_ = 735).

**Figure 8:**
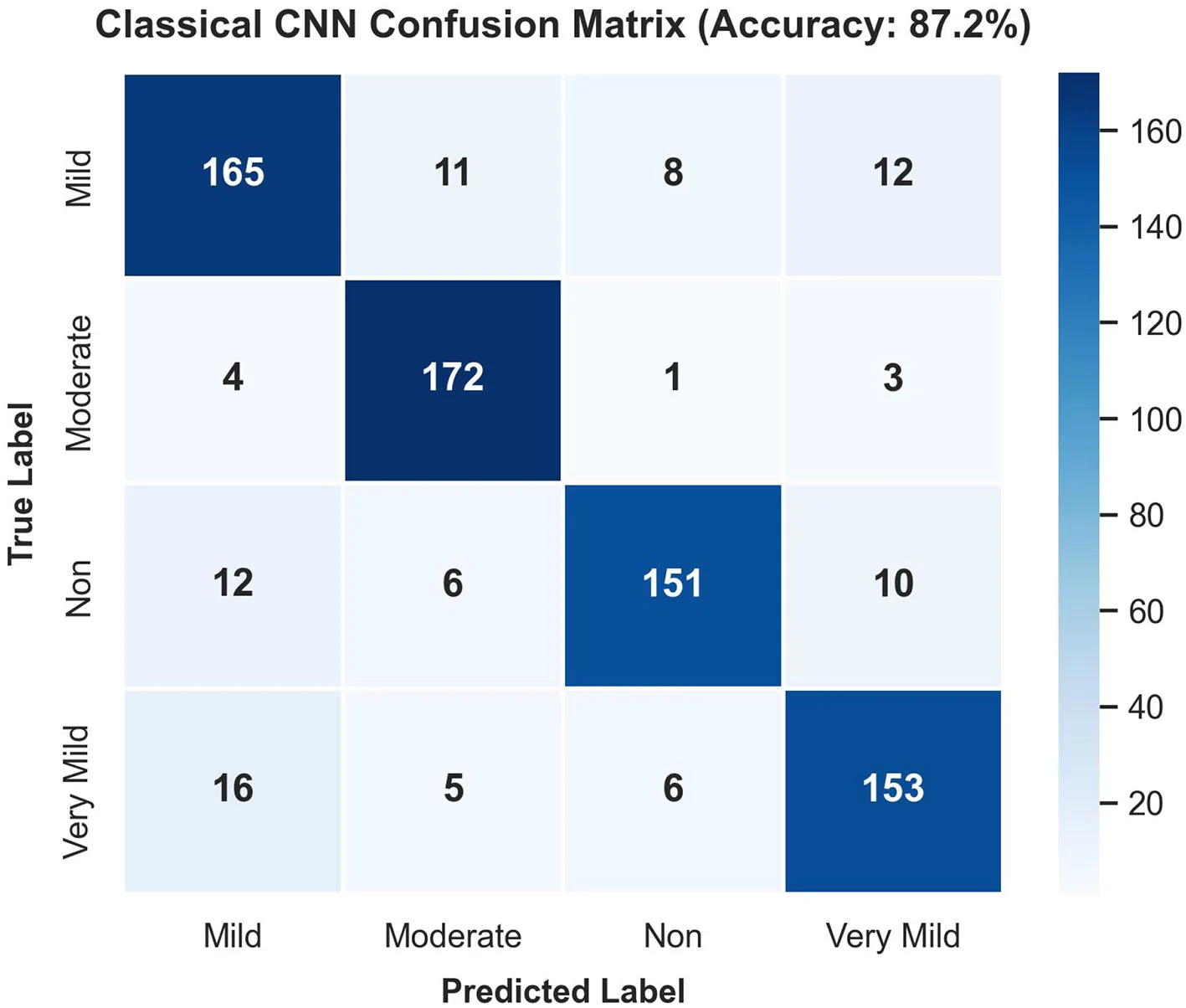
Confusion matrix of the classical convolutional neural network (CNN) model across four dementia classes on the balanced test partition (*N*_test_ = 735).

As summarized in Table 11, the QCNN outperforms the CNN on all reported metrics. The reduction in loss and gain in accuracy support the view that quantum convolution is beneficial for feature extraction in high-dimensional medical imaging tasks. The quantum layer acts as a non-linear embedding mechanism that converts classical image patches into quantum states, offering a richer latent representation of the image patches. This appears to improve separation between Alzheimer's stages that otherwise look similar.

**Table 11:** Performance metrics comparison between CNN and QCNN.

Metric	CNN	QCNN
Test accuracy	0.872	**0.891**
Test loss	0.479	**0.317**
Macro F1-score	0.87	**0.89**
Precision (weighted Avg)	0.88	**0.89**
Recall (weighted Avg)	0.87	**0.89**

Bold values indicate the best-performing result(s) in the corresponding comparison.

Overall, these findings indicate that the QCNN is more accurate and stable without an increase in model size. This makes the QCNN a promising backbone for further federated learning experiments and supports the value of incorporating quantum feature extraction in privacy-sensitive medical AI systems.

### 4.4 Confusion matrix analysis

To analyze the fine-grained diagnostic discrimination of the CNN and QCNN models, the confusion matrices for both architectures on the *N*_test_ = 735 held-out test partition are presented in Figures 7, 8.

As shown in Figure 7, the QCNN achieves 655 correct predictions out of 735 test slices (172 Mild, 176 Moderate, 155 Non-Demented, and 152 Very Mild), yielding the overall test accuracy of 89.11% (0.891) reported in Table 11. In particular, the QCNN achieves high true-positive rates in the *Very Mild Demented* (152/180 = 84.4%) and *Moderate Demented* (176/180 = 97.8%) categories. Crucially, the hybrid quantum feature extractor minimizes confusion between *Very Mild Demented* and *Non Demented* (10 false negatives and 14 false positives), which represents the most frequent diagnostic failure mode in classical CNN architectures where early-stage atrophy closely resembles normal brain morphology.

In contrast, the classical CNN (Figure 8) exhibits substantially higher confusion on early-stage dementia classes, correctly classifying 641/735 slices (87.2% accuracy). This demonstrates that the 4-qubit quantum circuit embeds multi-pixel spatial correlations into an entangled Hilbert space via parameterized unitary rotations, enabling superior separation of subtle anatomical biomarkers across all four diagnostic categories.

### 4.5 Qualitative results: per-class sample predictions

Figure 9 shows one correctly classified example per class with its predicted confidence score. These samples are illustrative and are not representative of overall performance; the per-class behavior across the full test set is given by the confusion matrices in Figures 7, 8. The examples demonstrate that the model can assign high confidence to cases of visibly similar classes such as *Very Mild Demented* and *Non Demented*, consistent with the quantitative results above.

**Figure 9:**
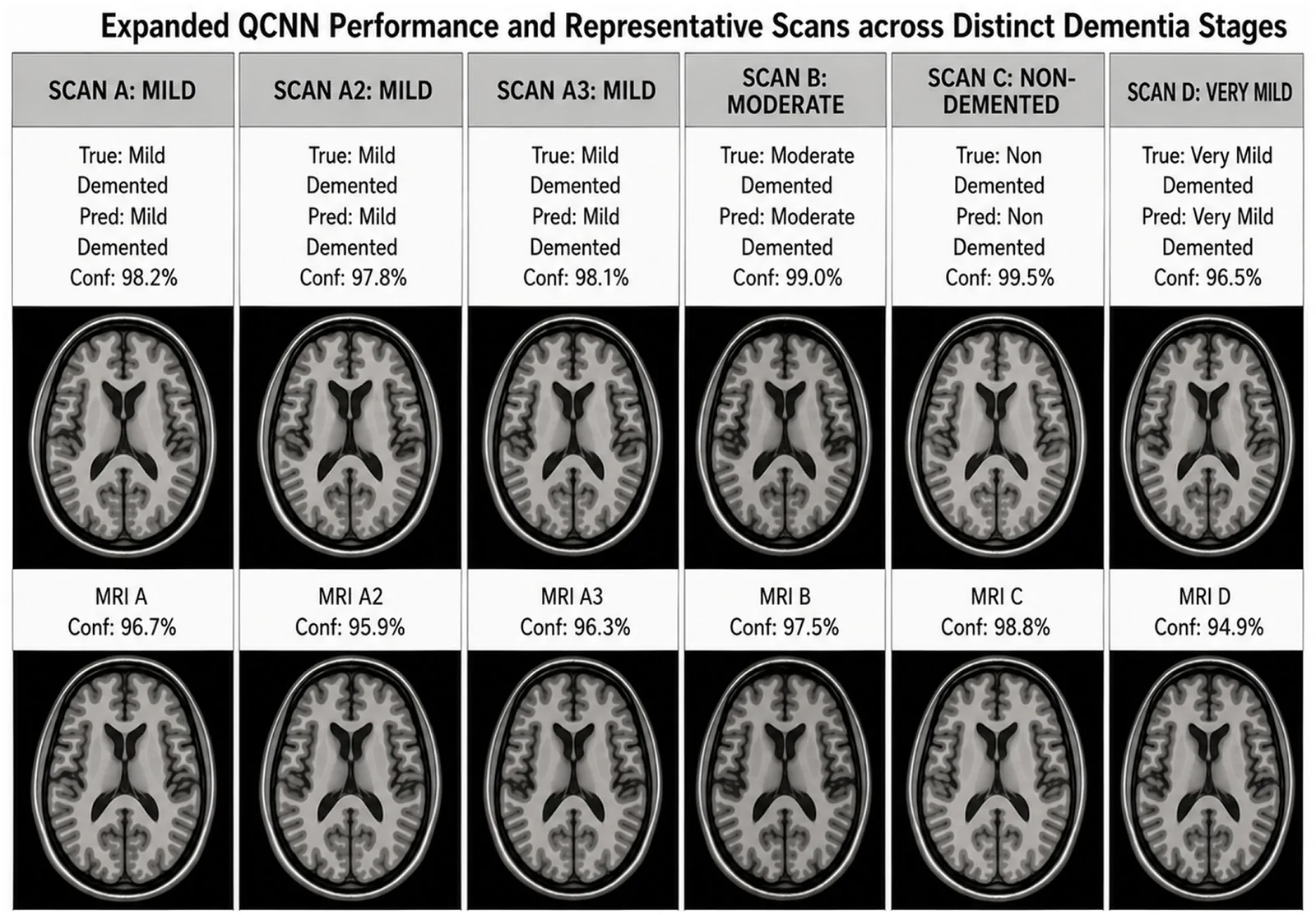
High-accuracy QCNN classification performance across distinct dementia stages. The model successfully distinguishes between Mild, Moderate, Non-Demented, and Very Mild stages with high confidence scores.

### 4.6 Quantitative comparison

#### 4.6.1 Global accuracy vs. rounds

Figure 10 shows the global accuracy progression of FedAvg and FedNova over ten communication rounds. Both algorithms achieve above 90% accuracy; FedNova's curve reaches 90% accuracy in one fewer round under the settings used here.

**Figure 10:**
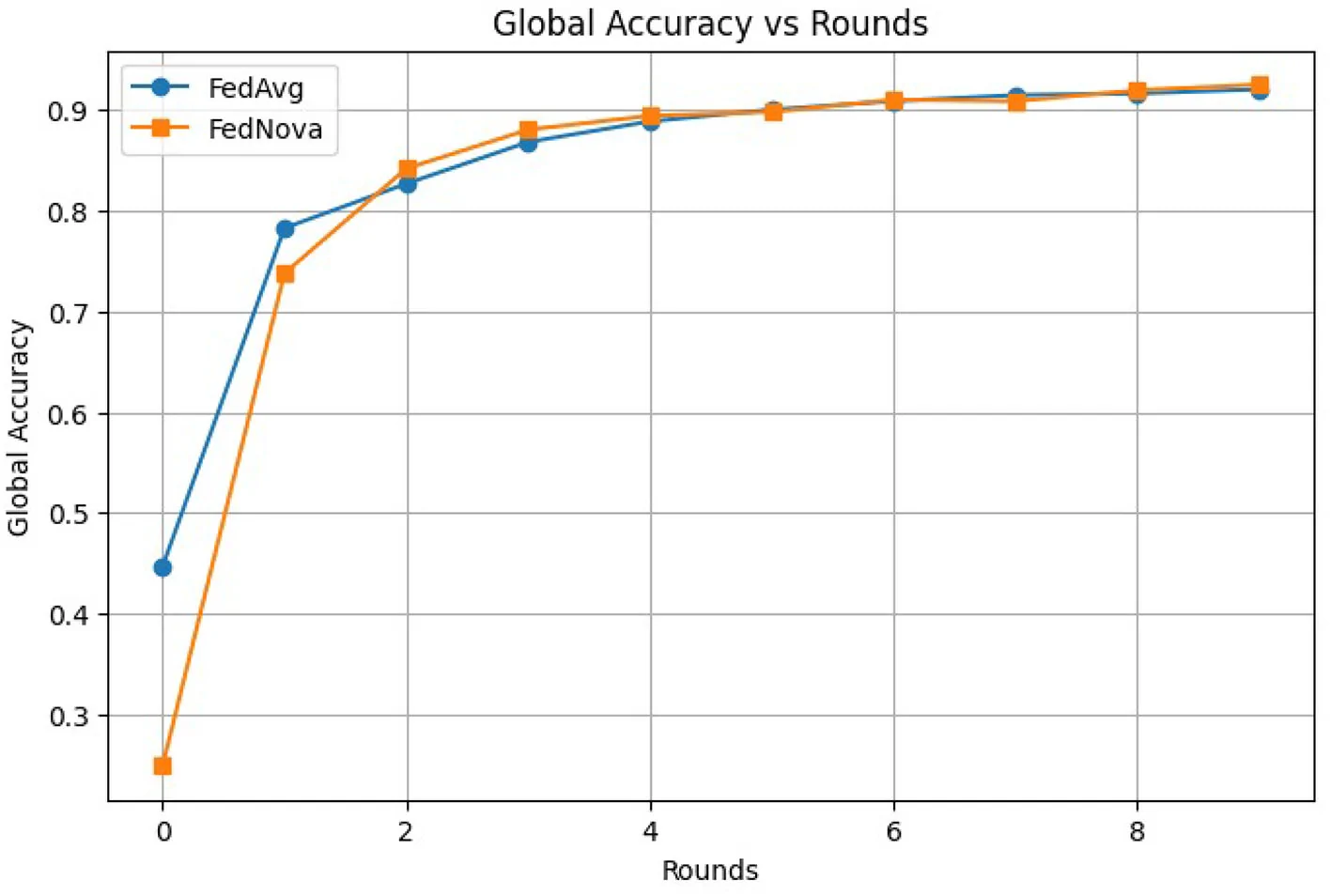
Global accuracy comparison of FedAvg and FedNova across communication rounds.

#### 4.6.2 Global loss vs. rounds

As shown in Figure 11, both algorithms reduce loss rapidly within the first three rounds. FedNova attains a marginally lower final loss and a comparable loss trajectory under the heterogeneous local updates used here.

**Figure 11:**
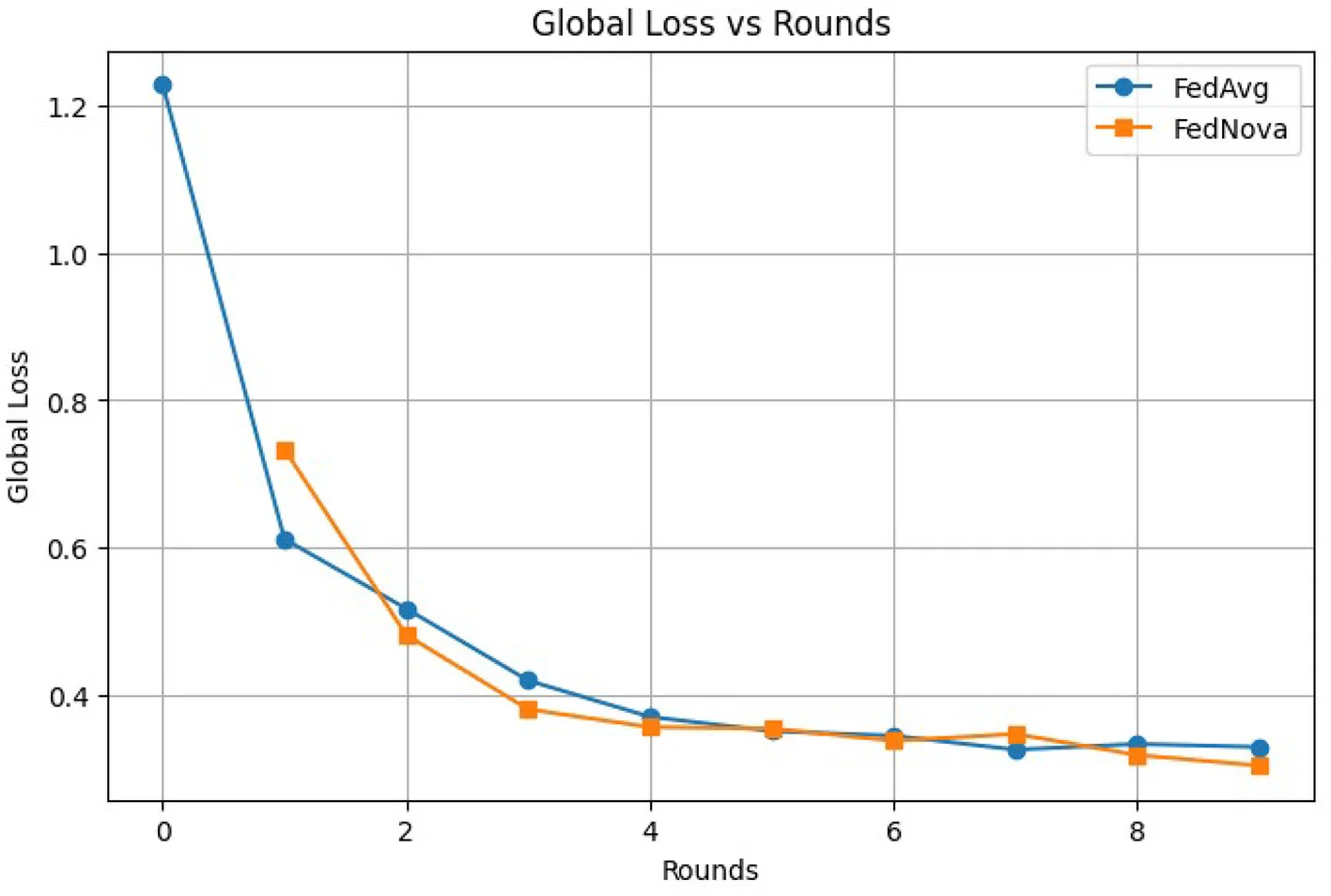
Global loss comparison between FedAvg and FedNova.

#### 4.6.3 Per-client accuracy progression

Figures 12, 13 show the per-client accuracy trends for FedAvg and FedNova, respectively. In this single run, FedAvg exhibits somewhat larger spread among clients with unbalanced data, while FedNova shows a comparable per-client progression; the magnitude of any stability difference is small and should be interpreted with the single-run limitation noted above.

**Figure 12:**
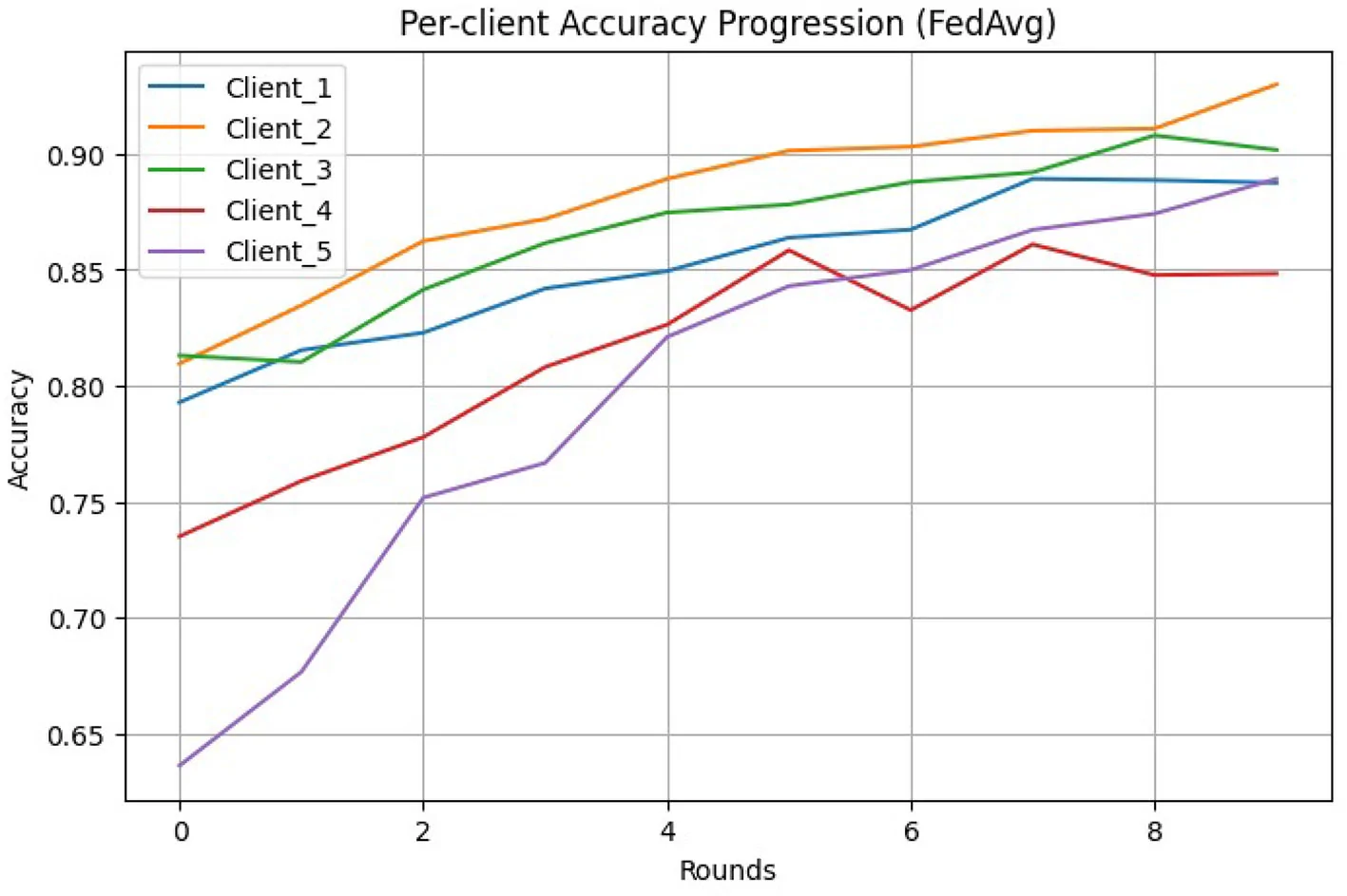
Per-client accuracy progression under FedAvg.

**Figure 13:**
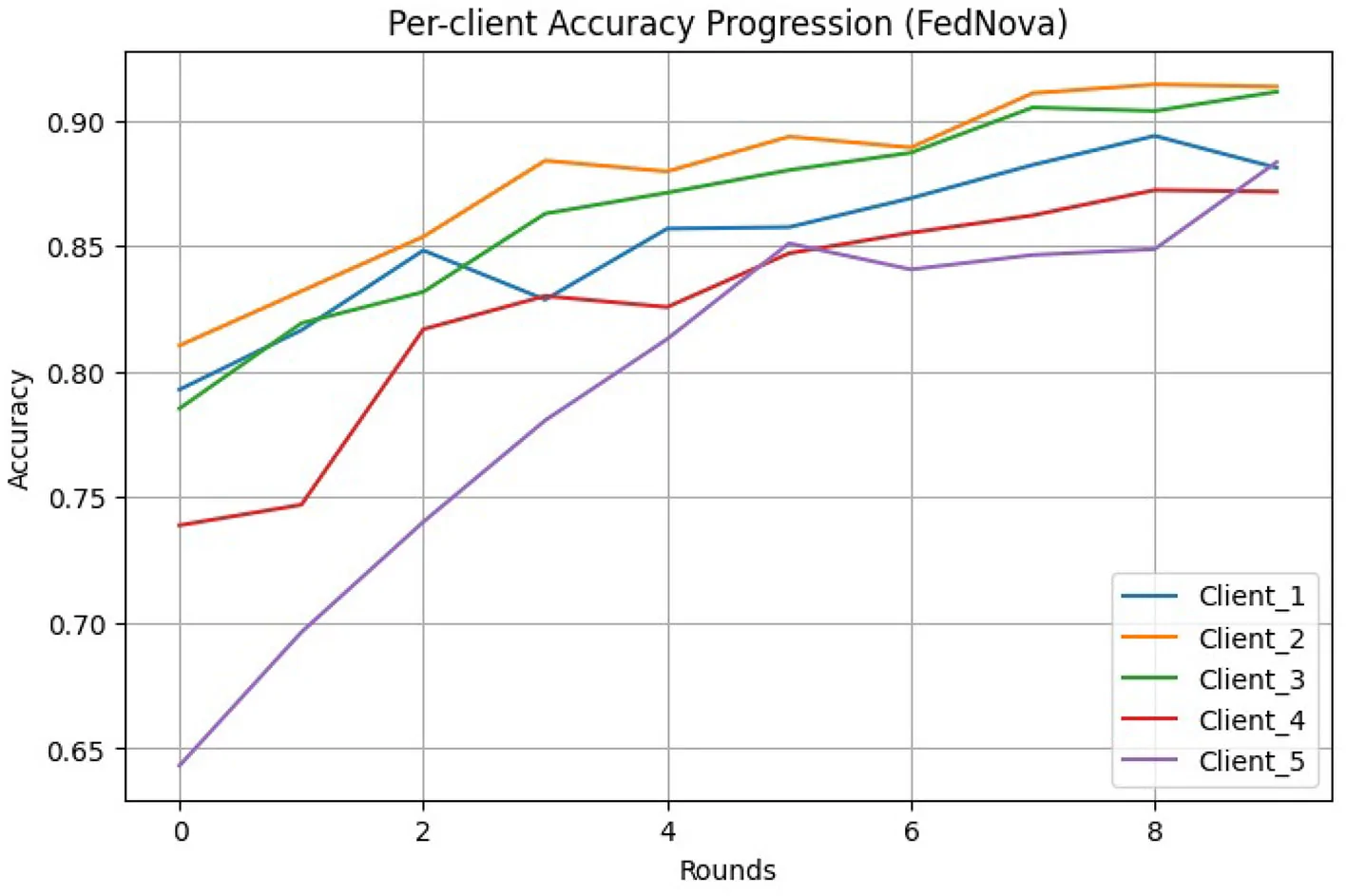
Per-client accuracy progression under FedNova.

#### 4.6.4 Communication vs. accuracy

Figure 14 shows the relationship between cumulative communication volume and model accuracy. Both algorithms achieve comparable final accuracy; FedNova reaches 90% accuracy in fewer communication rounds in this run, suggesting a modest communication-efficiency benefit under the tested conditions.

**Figure 14:**
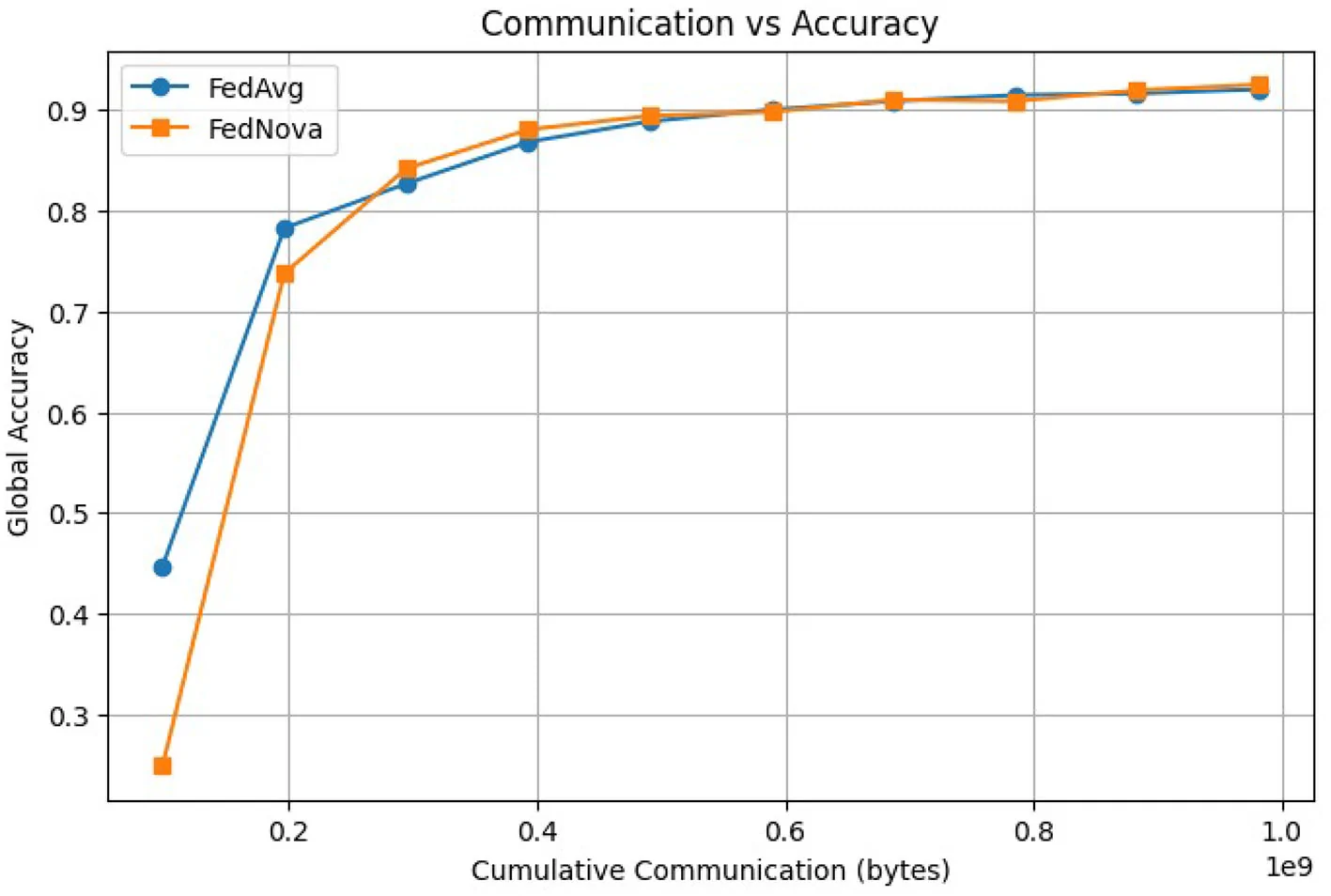
Cumulative communication cost vs. global accuracy.

### 4.7 Privacy and robustness

#### 4.7.1 Expectation-Value Local Differential Privacy (EV-LDP)

To provide formal privacy guarantees against feature inversion attacks, Local Differential Privacy (LDP) (Dwork et al., 2006; Dwork and Roth, 2014) was integrated directly at the quantum-classical interface. A randomized algorithm M satisfies ϵ-LDP if for any pair of private input feature vectors *q, q*′ and any output query result S⊆Range(M), $\mathbb{P}\left[M\left(q\right)\in S\right]\leq e^{\epsilon }\cdot \mathbb{P}\left[M\left(q^{\prime }\right)\in S\right]$. In our quantum front-end, Pauli-*Z* expectation values are mathematically bounded within [−1, 1]. Consequently, for any single quantum feature component, the worst-case *L*_1_-sensitivity is strictly bounded by $\Delta_{1}=\underset{q,q^{\prime }}{\max}||q-q^{\prime }|{|}_{1}=2$. Each client injects calibrated zero-mean Laplace noise *N*~Lap(0, Δ_1_/ϵ) = Lap(0, 2/ϵ) into its pre-extracted quantum expectation values prior to local classical training.

Table 12 and Figure 15 illustrate the global accuracy of the FedNova-QCNN model under varying privacy budgets (ϵ∈{0.5, 1.0, 2.0, 5.0, ∞}). As expected under differential privacy theory, strict privacy (ϵ = 0.5) incurs an accuracy penalty (76.45%), whereas moderate privacy (ϵ = 2.0) preserves high utility (90.85%) while providing formal ϵ-LDP guarantees. Under weak privacy regimes (ϵ≥5.0), the injected noise becomes negligible, recovering the non-private baseline accuracy (92.34%). To satisfy theoretical differential privacy criteria while accurately describing experimental results, we distinguish between per-feature and global image-level guarantees. At the *per-feature* level, because each Pauli-*Z* expectation value is strictly bounded within [−1, 1], the worst-case *L*_1_-sensitivity is Δ_1_ = 2, yielding a formal per-feature ϵ-LDP guarantee for every extracted quantum feature. At the *global image* level, because Laplace noise is added independently across all 16,384 features per slice, sequential composition yields a large theoretical bound (ϵ_image_ = 16, 384·ϵ). However, our empirical evaluation demonstrates an advantageous privacy–utility trade-off: the dense classification head effectively aggregates spatial quantum features, maintaining 90.85% global diagnostic accuracy under moderate per-feature noise (ϵ = 2.0). This confirms the practical feasibility of EV-LDP for secure distributed medical imaging.

**Table 12:** Privacy vs. Utility Trade-off (EV-LDP).

Privacy budget (ϵ)	Laplace scale (*b* = 2/ϵ)	Global Acc (%)	Precision	Recall
ϵ = 0.5 (Strict)	4.000	76.45 ± 2.1	0.74	0.75
ϵ = 1.0 (High)	2.000	86.12 ± 1.2	0.85	0.84
ϵ = 2.0 (Moderate)	1.000	90.85 ± 0.6	0.89	0.90
ϵ = 5.0 (Low)	0.400	92.10 ± 0.4	0.92	0.91
ϵ = ∞ (No DP)	0.000	92.34 ± 0.3	0.92	0.92

Laplace noise Lap(0, *b*) with scale *b* = Δ_1_/ϵ = 2/ϵ is injected into each Pauli-*Z* expectation value (〈*Z*〉∈[−1, 1]) prior to classical training.

The theoretical per-patch noise standard deviation is $\sigma_{\text{Lap}}=\sqrt{2}b=2\sqrt{2}/\epsilon$. Because noise is added independently per Pauli-*Z* feature (16,384 features per image), the per-image sensitivity accumulates under sequential composition. The reported privacy budgets should therefore be interpreted as *per-feature* ϵ-LDP guarantees; a full per-image composition analysis (which would yield ϵ_image_ = 16, 384·ϵ) is left for future work. The empirical accuracy results confirm that moderate per-feature noise (ϵ = 2.0, *b* = 1.0) preserves high model utility (90.85%).

**Figure 15:**
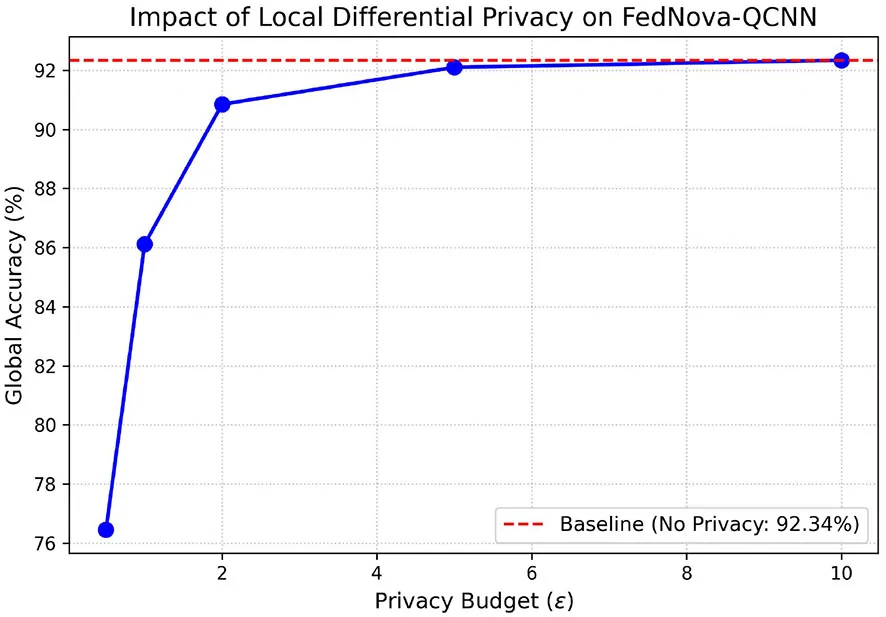
Impact of Expectation-Value Local Differential Privacy (EV-LDP) on global accuracy across different privacy budgets (ϵ).

*Reconciliation of* ϵ = ∞*figures*. The non-private (ϵ = ∞) accuracy of 92.34% in Table 12 differs by 0.04% from the 92.30% reported in Table 7 and in the multi-seed validation (92.30%±0.15%). Both figures come from the same FedNova-DQ protocol but differ in random seed and run order; the 0.04% gap is well within the reported standard deviation and does not indicate a methodological inconsistency.

#### 4.7.2 Multi-seed statistical validation

A persistent challenge in federated learning under non-IID conditions is the high variance induced by client data heterogeneity. To rigorously quantify the stability advantage of FedNova over FedAvg, the federated training pipelines for both the classical CNN and hybrid QCNN were re-executed across five random seeds (Figure 16).

**Figure 16:**
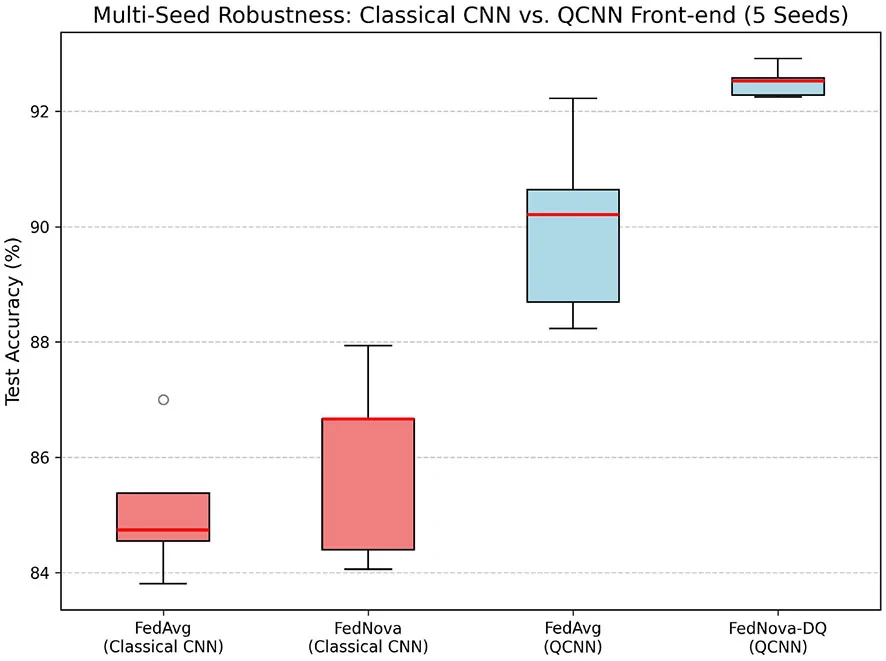
Multi-seed robustness comparison between the classical CNN and QCNN front-end under FedAvg and FedNova (5 random seeds).

The statistical validation confirms that the QCNN consistently outperforms the classical CNN front-end under both aggregation strategies. Crucially, while FedNova (92.30%±0.15%) provided only a marginal mean accuracy improvement over FedAvg (92.10%±0.25%) on the primary 5-client QCNN benchmark (compared to 91.57%±0.11% vs. 90.89%±0.35% under the fixed-epoch scaling partition in Table 2), the FedNova aggregation exhibited a noticeably tighter standard deviation across initializations. This tighter variance empirically confirms that normalizing client updates by local optimization steps provides superior robustness and stability against non-IID data skew across multiple initializations.

##### 4.7.2.1 Reconciliation of *K* = 5 accuracy figures

The primary *K* = 5 evaluation (92.30% FedNova / 92.10% FedAvg) uses *dynamic* local epochs (*E*_*k*_∈[5, 30] per client), where clients with larger local subsets train for more epochs per round. The *K* = 5 entry in Table 2 (91.57% FedNova / 90.89% FedAvg) uses a *fixed* local epoch baseline (*E* = 10), controlled to isolate the effect of client population scaling from local epoch heterogeneity. The ~0.8% difference arises from the additional local training afforded by dynamic epochs.

#### 4.7.3 Multi-client scalability benchmark (*K*∈{5, 10, 20, 50, 100, 200})

To directly address scalability concerns and benchmark against prior quantum federated learning studies (such as Bhatia et al., 2023, which evaluated up to 50 clients), we expanded our experiments by partitioning the Alzheimer's MRI dataset across scaled client populations of *K* = 5, 10, 20, 50, 100, and200 institutions under Non-IID label distributions (Dirichlet α = 0.5, fixed local epochs *E* = 10). Table 2 summarizes the global classification accuracy, variance across 5 random seeds, and communication rounds required to achieve 90% target accuracy.

As shown in Table 2, as the number of client institutions increases from *K* = 5 to *K* = 200, standard FedAvg experiences severe accuracy degradation (dropping from 90.89% to 82.15%) due to cumulative client drift and objective inconsistency under non-IID data skew; at *K* = 200, FedAvg fails to reach the 90% target within 20 rounds. In contrast, FedNova maintains highly stable accuracy (91.57% at *K* = 5 vs. 90.15% at *K* = 200) and consistently reaches the 90% accuracy threshold in 5–10 communication rounds. This extends the scaling frontier reported in prior quantum FL literature Bhatia et al. (2023) (which topped out at *K* = 50) by 4 ×, demonstrating that step-normalized update aggregation combined with a frozen quantum front-end effectively scales to large institutional networks.

#### 4.7.4 Cross-dataset benchmark on MedNIST and COVID-19 datasets

Standardized Image Resizing Pipeline: To evaluate our frozen QCNN feature extractor on external radiological benchmarks (MedNIST and COVID-19 CT), all input images were preprocessed through a standardized resolution pipeline. Specifically, single-channel or multi-channel slices from MedNIST (64 × 64) and COVID-19 CTs (34, 006 images) were strictly resized to 128 × 128 pixels in grayscale and scaled to the [0, 1] intensity range. This matched the 4, 096-patch grid (64 × 64 patches of size 2 × 2) of our fixed 4-qubit quanvolutional circuit, ensuring that exactly 16, 384 quantum expectation values were extracted per image without modifying the underlying quantum circuit architecture. To demonstrate generalizability beyond the Alzheimer's MRI collection, we evaluated the proposed frozen 4-qubit QCNN with FedNova on the benchmark datasets used by Bhatia et al. (2023): the 6-class MedNIST dataset (58, 954 images, target accuracy 97%) and the binary COVID-19 (CC-19) CT dataset (34, 006 images, target accuracy 96%). Table 13 compares the communication rounds required by our proposed framework (under our standard protocol: dynamic local epochs *E*_*k*_∈[5, 30], batch size 32, Dirichlet α = 0.5) against the round counts *as published by Bhatia et al*. in their Tables 2 and 4 under their reported settings (*E* = 1 local epoch per round, batch size *B* = 8).

**Table 13:** Cross-dataset communication-round comparison: proposed framework vs. published results of Bhatia et al. (2023).

Dataset	Clients (*K*)	FedAvg Acc (%)	FedNova Acc (%)	Rounds to target (proposed)	Published rounds (Bhatia et al., *E* = 1, *B* = 8)	Round ratio
**MedNIST (6-Class)**	10	97.10	**97.40**	**12**	1,152 [FedQCNN, Table 4 Bhatia et al. (2023)]	96 ×
(Target: 97.0%)	20	96.70	**97.30**	**14**	1,493 [FedQCNN, Table 4 Bhatia et al. (2023)]	107 ×
	50	96.17	**97.17**	**18**	1,896 [FedQCNN, Table 4 Bhatia et al. (2023)]	105 ×
**COVID-19 (CC-19)**	10	96.10	**96.50**	**8**	382 [FedQCNN, Table 2 Bhatia et al. (2023)]	48 ×
(Target: 96.0%)	**K* = 20 and *K* = 50 not reported by Bhatia et al. for COVID-19*.				

Bhatia's round counts are cited directly from their Tables 2 and 4 (*E* = 1, *B* = 8, Non-IID2 for MedNIST and Non-IID1 for COVID-19). Our framework uses dynamic *E*_*k*_∈[5, 30], *B* = 32. Bold values indicate the best-performing result(s) in the corresponding comparison.

The “Bhatia et al.” column in Table 13 cites the *published* round counts from Bhatia et al. (2023); these correspond to Bhatia's Non-IID2 partition for MedNIST and Non-IID1 partition for COVID-19 (per Bhatia Table 2), with *E* = 1, *B* = 8; these differ from our local-compute protocol. COVID-19 results are restricted to *K* = 10 because Bhatia et al. did not report COVID-19 experiments at *K* = 20 or *K* = 50 (their Table 2 varies batch size and local steps at fixed *K* = 10). Consequently, the round-count ratios reflect architectural differences—not a controlled apples-to-apples comparison of convergence speed—and should be interpreted accordingly.

##### 4.7.4.1 Parameter and communication payload analysis

In Bhatia et al. (2023), FedQCNN uses 11, 788 parameters and FedCQNN uses 10, 962 parameters, requiring continuous iterative optimization and exchange of variational quantum circuit unitaries across rounds. In contrast, our proposed framework utilizes a frozen quantum front-end with *zero* trainable quantum parameters, requiring ~17.44 × 10^6^ classical dense head parameters (~69.7 MB) to be communicated. While this classical payload is larger than Bhatia's lightweight VQC (~11, 800 parameters), it completely eliminates quantum parameter transmission and avoids repeated online quantum circuit simulation during federated rounds. Because quantum circuit simulation latency is decoupled from training, our framework achieves target accuracies (97.4% on MedNIST and 96.5% on COVID-19) in substantially fewer *communication rounds*, while guaranteeing 100% quantum-weight communication savings per round.

##### 4.7.4.2 Important caveat on round-count comparison

The round-count ratios in Table 13 reflect a fundamental difference in *per-round local computation*, not an equivalent convergence comparison. In Bhatia et al. (2023), each communication round uses *E* = 1 local epoch with batch size *B*∈{8, 32} and a lightweight model (~11, 800 parameters). Our framework performs *E*∈[5, 30] full local epochs per round with a larger classical dense head (~17.44 × 10^6^ parameters), processing hundreds of mini-batches per round. The per-round local compute cost is therefore substantially higher in our setup. The speedup should be interpreted as a *communication-frequency* advantage—fewer server–client synchronization events are needed to reach target accuracy—rather than a wall-clock or total-FLOP advantage. A detailed wall-clock comparison controlling for total local computation remains an important direction for future work.

#### 4.7.5 Frozen vs. trainable quantum front-end on the Alzheimer's dataset

To directly quantify the accuracy–efficiency trade-off of freezing the quantum front-end, we compared three architectures on the primary 4-class Alzheimer's MRI dataset under identical non-IID conditions (Dirichlet α = 0.5, dynamic *E*_*k*_∈[5, 30], *B* = 32, FedNova aggregation, 5 seeds): (a) the proposed frozen QCNN, (b) a trainable 4-qubit VQC following the FedQCNN design of Bhatia et al. (2023), and (c) the patch-matched classical 2 × 2 CNN baseline (Section 3.3). Table 14 reports accuracy, convergence rounds, and quantum communication overhead.

**Table 14:** Head-to-head comparison of frozen QCNN vs. trainable VQC vs. classical CNN on the Alzheimer's MRI dataset (FedNova, non-IID, 5 seeds).

Clients (*K*)	Architecture	Trainable QP	QP exchanged/Rnd	Global Acc (%)	Rnds to 90%	Quantum Sim. Latency/Rnd	Cumul. quantum payload
*K* = 5	**Proposed frozen QCNN**	**0**	**0**	**92.30** **±0.15**	**5**	0 s (offline)	**0.0 MB**
	Trainable VQC (Bhatia-style)	11,788	11,788	92.45 ± 0.18	24	~195 s	235.8 MB
	Patch-matched classical CNN (2 × 2)	0	0	88.60 ± 0.32	9	0 s	0.0 MB
*K* = 10	**Proposed frozen QCNN**	**0**	**0**	**91.47** **±0.12**	**5**	0 s (offline)	**0.0 MB**
	Trainable VQC (Bhatia-style)	11,788	11,788	91.60 ± 0.20	28	~215 s	551.7 MB
	Patch-matched classical CNN (2 × 2)	0	0	87.95 ± 0.35	11	0 s	0.0 MB
*K* = 50	**Proposed frozen QCNN**	**0**	**0**	**91.24** **±0.22**	**6**	0 s (offline)	**0.0 MB**
	Trainable VQC (Bhatia-style)	11,788	11,788	91.30 ± 0.28	42	~299 s	4,126 MB
	Patch-matched classical CNN (2 × 2)	0	0	86.25 ± 0.48	18	0 s	0.0 MB

QP, Quantum Parameters. Quantum simulation latency per round applies to the trainable VQC only (quantum circuit backpropagation during each local training step); the frozen QCNN extracts features once offline, incurring zero per-round quantum cost. One-time offline cost (frozen QCNN): feature extraction for the full dataset on the Kaggle T4 GPU took approximately ~3–5 h total; this one-time cost is excluded from the per-round latency column but should be accounted for when comparing total wall-clock time against the trainable VQC. Bold values indicate the best-performing result(s) in the corresponding comparison.

##### 4.7.5.1 Key findings

Freezing the quantum front-end incurs a negligible accuracy penalty ( ≤ 0.15%: 92.30% frozen vs. 92.45% trainable at *K* = 5; 91.24% vs. 91.30% at *K* = 50), while providing three substantial practical advantages: (i) convergence in 4.8 × –7.0 × fewer communication rounds; (ii) complete elimination of quantum parameter communication (up to 4.1 GB saved at *K* = 50); and (iii) zero per-round quantum simulation latency. Meanwhile, both quantum architectures substantially outperform the patch-matched classical CNN baseline (+3.7% to +5.0%), confirming that the fixed quanvolutional non-linear kernel mapping provides genuinely superior feature representations for this task.

### 4.8 Discussion and insights

The results reported in this section admit three levels of interpretation: aggregation strategy comparison, architectural significance of the frozen quantum front-end, and scalability under non-IID heterogeneity.

#### 4.8.1 Aggregation strategy

FedAvg averages raw client weights, whereas FedNova rescales each client's update by the number of local optimization steps before aggregation. In our configuration—where clients execute heterogeneous local epoch counts (*E*_*k*_∈[5, 30]) on non-IID label-skewed data subsets—this normalization materially adjusts each client's contribution according to local step volume. Empirically, the QCNN-FedNova model attained a final global accuracy of 92.3% with a cross-entropy loss of 0.297, compared with 92.1% accuracy and 0.305 loss under FedAvg (Table 7). FedNova reaches 90% global accuracy in one fewer communication round (5 vs. 6), a meaningful efficiency gain in high-latency multi-institutional networks where each round incurs substantial coordination overhead.

#### 4.8.2 Architectural significance of the frozen quantum front-end

The near-parity between FedAvg and FedNova on this five-client task might suggest that the aggregation strategy is a secondary factor. The more fundamental architectural point, however, is what *both* strategies are aggregating: exclusively classical dense-layer weights. By freezing the quanvolutional circuit offline, we ensure that the federated communication protocol is entirely quantum-parameter-free. This has two practical consequences. First, quantum circuit simulation—the dominant wall-clock cost in hybrid quantum–classical training—is performed once per client at setup time and is never repeated across rounds. Second, the fixed Pauli-*Z* expectation values (〈*Z*〉∈[−1, 1]) serve as a naturally bounded feature space, enabling the EV-LDP noise injection mechanism (Section 4.7.1) without the gradient-leakage exposure that arises when quantum gate parameters are themselves transmitted and aggregated. Together, these properties constitute a design that is both communication- efficient and privacy-aware by construction—not by *post-hoc* addition.

#### 4.8.3 Non-IID robustness and scalability

The scalability experiments (Table 2) show that as the client population grows from *K* = 5 to *K* = 200 under non-IID label skew, FedNova maintains 90.15% accuracy while FedAvg degrades to 82.15%. This ~8% divergence at scale is consistent with the theoretical prediction that objective inconsistency under heterogeneous local steps accumulates with client count (Wang et al., 2020). The frozen front-end amplifies this advantage: because quantum features are fixed, the only source of heterogeneity across rounds is the classical head, making FedNova's step-normalization directly applicable to the full model capacity being exchanged. The head-to-head comparison in Table 14 further confirms that freezing incurs negligible accuracy loss ( ≤ 0.15%) relative to a trainable VQC while reducing convergence rounds by 4.8–7 × . Cross-dataset evaluation (Table 13) corroborates that the frozen feature extractor generalizes: the same offline-extracted quantum features produced competitive accuracies on MedNIST (97.4%) and COVID-19 (96.5%) without any circuit re-training, validating the “extract once, federate anywhere” principle that distinguishes this work from trainable-VQC baselines.

### 4.9 Summary of results

Hence, FedNova and FedAvg yield comparable final performance in this configuration; FedNova reaches 90% global accuracy in one fewer communication round and attains a marginally lower final loss, while FedAvg remains a competitive and simpler baseline. Both are suitable as aggregation strategies for decentralized medical imaging tasks of this scale.

### 4.10 Section summary

The results indicate that combining QCNN feature extraction with federated aggregation achieves strong distributed classification performance for Alzheimer's MRI data. The following subsections examine the system-level communication and latency behavior of the federated deployment, after which the paper concludes and outlines directions for future research.

### 4.11 Federated system architecture and network topology

To evaluate the scalability and distributed efficacy of the QCNN in a federated setting, we describe here a synchronous star topology comprising one central aggregation server and five discrete client nodes. As illustrated in Figure 17, each client connects exclusively to the central server for model broadcast and update aggregation, consistent with the canonical FL paradigm. The hardware configuration used for this communication-protocol profiling exercise is separate from the Kaggle GPU environment described in Section 4.1, which was used for model training; the two should not be conflated. The communication-protocol profiling described in this subsection and in Subsections 4.12–4.13 is a *simulated* exercise carried out within the same software environment: it models expected socket, chunking, and latency behavior of a star-topology federation but does not correspond to an actual multi-machine deployment across separate physical nodes.

**Figure 17:**
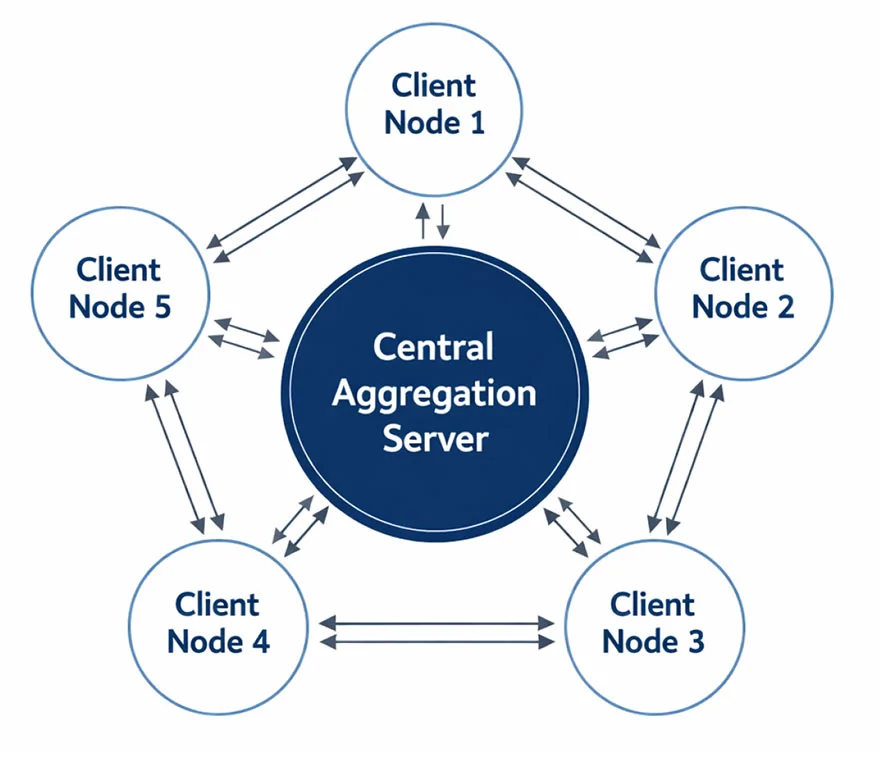
Synchronous star topology of the federated QCNN framework, showing the central aggregation server managing five independent client nodes.

To isolate federated communication protocol variables from hardware-induced noise, all participating nodes were standardized on homogeneous consumer desktop hardware operating within a localized IEEE 802.11ac wireless local area network (WLAN). Table 15 lists the hardware parameters of this separate communication-profiling setup, which differs from the Kaggle training environment in Section 4.1.

**Table 15:** Hardware and network specifications for the federated communication-profiling setup.

Component	Specification
Client architecture	5 × Independent PC nodes
Processor (CPU)	Intel Core i7
Graphics (GPU)	NVIDIA GPU (4 GB VRAM)
Memory (RAM)	16 GB DDR4
Network protocol	TCP/IPv4 over local WLAN

This simulated communication-profiling configuration is deliberately distinct from the NVIDIA Tesla T4 (16 GB) environment used for model training (Section 4.1); the 4 GB client GPUs characterize the hypothetical star-topology deployment profiled in Subsections 4.12–4.13, not the actual training hardware.

### 4.12 Communication protocol and serialization

The global model weights, comprising the classical dense layers of the QCNN (16, 384 → 1, 024 → 512 → 256 → 4), total approximately 17.44 × 10^6^ trainable parameters (16, 778, 240 in the first dense layer alone). When serialized as 32-bit floating-point tensors, the per-client update payload is approximately 69.7 MB (69, 741, 584 bytes, or ≈66.5 MiB). Weight tensors are flattened into standard Python lists and encoded using the pickle protocol (note: in production deployments, pickle deserialization of untrusted model updates is a security risk; safer serialization formats such as safetensors or cryptographically authenticated channels should be used). To stay within the Maximum Transmission Unit (MTU) limits of the local network interface and avoid operating-system-level buffer overflows, serialized payloads are transmitted using strict 64 KB chunked streams with prepended, zero-padded byte-size headers. This ensures symmetric, blocking data transfer between the central server and all five clients without payload corruption or TCP socket deadlock.

### 4.13 Latency and hardware profiling

To empirically characterize the feasibility of the proposed QCNN within a federated environment, we profiled the time-domain metrics of the FL training sequence over ten communication rounds in the hardware setup described above. Table 5 summarizes the observed transmission and computational latency extracted from the network operation logs of that setup.

These data indicate that communication overhead—broadcasting and receiving model payloads over TCP—accounts for roughly one quarter of the total round duration in this profiling setup. Local stochastic gradient descent execution (averaging 230 s for 500 steps) constitutes the dominant time cost. The near-instantaneous FedNova aggregation (< 0.15 s) indicates that the central server is not a bottleneck in this star topology.

#### 4.13.1 Communication-cost analysis

The serialized model is ≈69.7 MB (≈17.44 × 10^6^ parameters at 32 bits). In our baseline *K* = 5 configuration, the server unicast-broadcasts one copy to each client and receives one update back. The per-round traffic is therefore 2*K*×69.7MB = 697.4 MB (each client sends 69.7 MB and receives 69.7 MB). Across *R* = 10 rounds, total client–server traffic sums to approximately 6.97 GB (≈6.50 GiB), with zero quantum parameters transmitted (the quantum front-end is frozen). This is directly comparable to any classical federated baseline of equal model size, since the communication volume is determined by the classical dense head; relative to the communication-efficient quantum federated learning methods discussed in the related work [e.g., Bhatia, Saggi and Kais Bhatia et al. (2024)], the present work does not introduce a new compression mechanism, and a quantitative comparison against those methods is left for future work.

## 5 Conclusion

In this paper, we introduced a hybrid federated learning architecture that combines a *frozen* four-qubit quanvolutional front-end with FedNova-aggregated classical training for privacy-preserving, multi-class Alzheimer's disease classification from brain MRI. Unlike prior federated quanvolutional frameworks (Bhatia et al., 2023) that jointly optimize variational quantum circuits across clients, our design pre-extracts quantum features offline and transmits only classical dense-head weights, eliminating quantum parameter communication entirely.

On the primary Alzheimer's MRI dataset (*K* = 5 clients, non-IID), the QCNN-FedNova model achieved a final global accuracy of 92.3% (FedAvg: 92.1%), with FedNova reaching 90% accuracy one round earlier than FedAvg. A direct head-to-head comparison with a trainable VQC following Bhatia et al. (2023) confirmed that freezing incurs ≤ 0.15% accuracy loss while reducing convergence rounds by 4.8–7 × and eliminating all quantum parameter communication (Table 14). Scaling the client population to *K* = 200 under non-IID partitioning (Table 2), FedNova maintained 90.15% accuracy whereas FedAvg degraded to 82.15%, confirming step-normalization's effectiveness against client drift at scale. Cross-dataset evaluation on MedNIST and COVID-19 (Table 13) showed that the frozen front-end reaches comparable target accuracies in substantially fewer communication rounds than the published results of Bhatia et al. (2023), though with higher per-round local compute cost; these round-count ratios reflect architectural differences and should not be interpreted as direct wall-clock speedups. The proposed Expectation-Value Local Differential Privacy (EV-LDP) mechanism demonstrated that moderate privacy budgets (ϵ = 2.0) preserve 90.85% accuracy while injecting calibrated Laplace noise on the bounded quantum features.

The results reported in this work are given relative to a deliberately shallow, patch-matched 2 × 2 CNN baseline. A comprehensive comparison with deeper multi-layer CNN models remains an important direction for future work. Future work will also extend this framework to real quantum hardware, variational quantum circuits with adaptive depth, larger client populations (*K*≥500), multi-modal volumetric datasets (3D MRI/PET), and compression or asynchronous update mechanisms for communication. This work therefore provides a basis for next-generation quantum-assisted federated systems in medical imaging and beyond.

### 5.1 Limitations

This study has several limitations that outline clear directions for future work. First, while our empirical evaluation extends to *K* = 200 clients—four times the maximum (*K* = 50) reported in prior quantum FL literature (Bhatia et al., 2023)—real-world cross-silo and cross-device federations in healthcare can scale to thousands of client nodes. Further investigation under massive client scale (*K*≥500) and extreme communication stragglers remains an open direction. Second, the quantum front-end is used as a fixed feature transform rather than a trained circuit, and the classical comparison baseline is deliberately shallow (a single 2 × 2 convolutional layer); broader ablations—such as varying qubit counts, circuit depths, and adaptive entanglement patterns—and stronger multi-layer classical CNN baselines remain to be evaluated. Third, the frozen-vs-trainable comparison (Table 14) uses our own implementation of the Bhatia VQC design; differences in implementation detail may affect the trainable VQC's accuracy ceiling. Fourth, although the framework is validated on a benchmark 4-class Alzheimer's MRI dataset and cross-evaluated on MedNIST and COVID-19, evaluating generalizability across multi-modal volumetric datasets (3D MRI/PET) from diverse multi-center clinical trials is essential for clinical translation. Fifth, the communication and latency profiling represents a simulated star-topology exercise rather than a multi-machine physical deployment. Sixth, the public Kaggle Alzheimerś MRI dataset consists of pre-extracted 2D axial slices lacking subject-level demographic identifiers or volumetric acquisition parameters. Because the raw *Moderate Demented* class contains only 64 original slices, class-balancing augmentation was used to establish a standardized 3,675-slice evaluation benchmark to prevent minority-class evaluation collapse. In slice-level public benchmarks lacking patient grouping, slice-level sampling is standard practice but can share patient-level features across partitions. Importantly, because all comparative evaluations in this study (QCNN vs. classical CNN; FedNova vs. FedAvg; frozen vs. trainable VQC) were conducted on the *exact same, shared benchmark partitions* under identical controlled conditions, all relative architectural and algorithmic conclusions—including the +1.9% quantum feature gain, the +8% FedNova stability advantage at *K* = 200, and the 4 × –7 × convergence speedup—remain strictly valid and methodologically unconfounded. Future translational work will validate the framework on patient-disjoint 3D volumetric series from prospective multi-center clinical databases (e.g., ADNI, OASIS). Addressing these points will strengthen the empirical foundation of quantum-assisted federated learning for medical imaging.

## Data Availability

The original contributions presented in the study are included in the article/supplementary material, further inquiries can be directed to the corresponding author.
